# Financial and Economic Costs of the Elimination and Eradication of Onchocerciasis (River Blindness) in Africa

**DOI:** 10.1371/journal.pntd.0004056

**Published:** 2015-09-11

**Authors:** Young Eun Kim, Elisa Sicuri, Fabrizio Tediosi

**Affiliations:** 1 Swiss Tropical and Public Health Institute, Basel, Switzerland; 2 University of Basel, Basel, Switzerland; 3 ISGlobal, Barcelona Centre for International Health Research (CRESIB), Hospital Clínic - Universitat de Barcelona, Barcelona, Spain; Imperial College London, School of Public Health, UNITED KINGDOM

## Abstract

**Background:**

Onchocerciasis (river blindness) is a parasitic disease transmitted by blackflies. Symptoms include severe itching, skin lesions, and vision impairment including blindness. More than 99% of all cases are concentrated in sub-Saharan Africa. Fortunately, vector control and community-directed treatment with ivermectin have significantly decreased morbidity, and the treatment goal is shifting from control to elimination in Africa.

**Methods:**

We estimated financial resources and societal opportunity costs associated with scaling up community-directed treatment with ivermectin and implementing surveillance and response systems in endemic African regions for alternative treatment goals—control, elimination, and eradication. We used a micro-costing approach that allows adjustment for time-variant resource utilization and for the heterogeneity in the demographic, epidemiological, and political situation.

**Results:**

The elimination and eradication scenarios, which include scaling up treatments to hypo-endemic and operationally challenging areas at the latest by 2021 and implementing intensive surveillance, would allow savings of $1.5 billion and $1.6 billion over 2013–2045 as compared to the control scenario. Although the elimination and eradication scenarios would require higher surveillance costs ($215 million and $242 million) than the control scenario ($47 million), intensive surveillance would enable treatments to be safely stopped earlier, thereby saving unnecessary costs for prolonged treatments as in the control scenario lacking such surveillance and response systems.

**Conclusions:**

The elimination and eradication of onchocerciasis are predicted to allow substantial cost-savings in the long run. To realize cost-savings, policymakers should keep empowering community volunteers, and pharmaceutical companies would need to continue drug donation. To sustain high surveillance costs required for elimination and eradication, endemic countries would need to enhance their domestic funding capacity. Societal and political will would be critical to sustaining all of these efforts in the long term.

## Author Summary

River blindness (onchocerciasis) is a parasitic disease transmitted by blackflies. Symptoms include severe itching, skin lesions, and vision impairment including blindness. More than 99% of all cases are concentrated in sub-Saharan Africa. Fortunately, vector control and community-directed treatment with ivermectin have significantly decreased morbidity, and the treatment goal is shifting from control to elimination in Africa. To inform policymakers’ and donors’ decisions, we estimated financial resources and societal opportunity costs associated with alternative treatment goals—control, elimination, and eradication. We found that rapid scale-up of ivermectin treatment for elimination and eradication would result in substantial cost-savings in the long term as compared to staying in a control mode, because regular active surveillance would allow treatments to end earlier, thereby saving the economic costs of community volunteers and donated ivermectin. To realize cost-savings, policymakers should keep empowering community volunteers, and pharmaceutical companies would need to continue drug donation. To sustain high surveillance costs required for elimination and eradication, endemic countries would need to enhance their domestic funding capacity. Societal and political will would be critical to sustaining all of these efforts.

## Introduction

The treatment goal for onchocerciasis (river blindness) has shifted from control to elimination as shown by the World Health Organization’s (WHO’s) roadmap for neglected tropical diseases (NTDs) and the London Declaration on NTDs in 2012 [1,2]. Onchocerciasis is a parasitic disease transmitted by blackflies, and notable symptoms include severe itching, skin lesions, and vision impairment including blindness. Those affected by onchocerciasis suffer negative socioeconomic consequences as a result of their symptoms [3]. The disease is endemic in parts of Africa, Latin America, and Yemen, and more than 99% of all cases are concentrated in sub-Saharan Africa [4]. In Africa, morbidity caused by onchocerciasis was significantly reduced by the vector control activities of the Onchocerciasis Control Programme (OCP) in West Africa (1975–2002) and by the community-directed treatment with ivermectin (CDTi) under the African Programme for Onchocerciasis Control (APOC) in sub-Saharan Africa and parts of West Africa (1995–present) [4]. Studies of foci in Mali, Senegal, and Uganda have proved that eliminating onchocerciasis through ivermectin administration is feasible for amenable epidemiological settings under effective treatments and surveillance [5,6].

Onchocerciasis elimination and subsequent eradication will generate health benefits by reducing the incidence of infection to zero, first in a defined area and then globally. These benefits would be higher than those of staying in a control mode that keeps disease prevalence at a locally acceptable level. In addition to epidemiological evidence, national and global policymakers must consider economic, social, and political aspects when deciding whether to invest in elimination in settings with limited resources and competing health priorities. To assess these broad aspects, a working group at the Ernst Strüngmann Forum suggested developing and analyzing eradication/elimination investment cases [7]. Tediosi and colleagues examined the suggested approach focusing on three NTDs including onchocerciasis [8]. Referring to this study, Kim and colleagues defined investment options for onchocerciasis as scenarios, and compared the respective timelines and needs for treatment in endemic African countries [9]. Each scenario consists of strategies of treatments and surveillance—epidemiological surveillance to track the infection levels in human and/or entomological surveillance to track the infectivity rates of blackflies.

### Control scenario

To reduce disease prevalence to a locally acceptable level (i.e., microfilaria prevalence≤40% or community microfilarial load≤5mf/s [3]), all endemic African countries implement annual CDTi in hyper- and meso-endemic areas, and after at least 25-years of CDTi, conduct epidemiological surveillance to confirm that CDTi can be safely stopped (former OCP projects having implemented regular surveillance continue their surveillance strategies).

### Elimination scenario

To reduce the incidence of infection to zero in a defined area, all endemic African countries except those with epidemiological and political challenges implement annual or biannual CDTi, and conduct regular active epidemiological and entomological surveillance to evaluate epidemiological trends, to decide a proper time to stop CDTi, and to detect and respond to possible recrudescence.

### Eradication scenario

To reduce the incidence of infection to zero in Africa, which would lead to global eradication, all endemic African countries implement not only annual or biannual CDTi but also locally tailored treatment strategies to deliver sustainable treatments to areas with operational challenges, and implement regular active epidemiological and entomological surveillance to evaluate epidemiological trends, to decide a proper time to stop CDTi, and to detect and respond to possible recrudescence.

We estimated financial resources and societal opportunity costs for endemic African countries (Table 1) associated with the control, elimination, and eradication scenarios to support policymakers’ and donors’ informed decisions and provide a basis for further economic evaluation of the elimination and eradication of onchocerciasis.

**Table 1:** Endemic African countries: GDP per capita, health expenditure (total, out of pocket), population living in endemic areas.

Country	Program	GDP per capita, 2012	Total health expenditure (THE), 2012 (% of GDP)	Out-of-pocket health expenditure, 2012 (% of THE)	Population living in endemic areas, 2014
Angola	APOC	$5,539	3.47%	26.69%	2,640,000
Benin	former OCP	$751	4.49%	44.26%	3,585,000
Burkina Faso	former OCP	$652	6.17%	36.36%	230,000
Burundi	APOC	$251	8.13%	28.27%	1,613,000
Cameroon	APOC	$1,220	5.13%	62.65%	9,040,000
Central African Rep.	APOC	$479	3.76%	45.57%	2,150,000
Chad	APOC	$1,035	2.81%	66.43%	2,182,000
Congo, Dem. Rep.	APOC	$418	5.59%	32.48%	43,633,000
Congo, Rep.	APOC	$3,154	3.16%	25.07%	1,475,000
Côte d’Ivoire	former OCP	$1,244	7.06%	55.83%	2,359,000
Equatorial Guinea	APOC	$22,391	4.74%	43.53%	88,000
Ethiopia	APOC	$467	3.83%	41.22%	12,276,000
Gabon	APOC	$10,930	3.47%	41.41%	85,000
Ghana	former OCP	$1,646	5.17%	28.72%	2,535,000
Guinea	former OCP	$493	6.30%	66.62%	3,332,000
Guinea-Bissau	former OCP	$494	5.86%	43.18%	195,000
Liberia	APOC	$414	15.53%	21.22%	3,169,000
Malawi	APOC	$267	9.16%	12.58%	2,261,000
Mali	former OCP	$696	5.82%	60.73%	5,146,000
Mozambique	APOC	$570	6.42%	5.04%	67,000
Nigeria	APOC	$2,742	6.07%	65.88%	55,255,000
Senegal	former OCP	$1,023	4.96%	34.14%	187,000
Sierra Leone	former OCP	$633	15.08%	76.23%	3,320,000
South Sudan	APOC	$974	2.55%	56.70%	7,307,000
Sudan	APOC	$1,695	7.25%	73.68%	657,000
Tanzania	APOC	$609	6.99%	31.75%	3,536,000
Togo	former OCP	$589	8.64%	41.08%	3,172,000
Uganda	APOC	$551	7.97%	49.33%	4,473,000
Average (SD)		$2,212 ($4,507)	6.27% (3.10%)	43.45% (18.25%)	6,285,000 (12,604,000)

GDP per capita, 2012 (USD 2012) from World Bank [10];

Total health expenditure (THE), 2012 (% of GDP) from World Bank [11];

Out-of-pocket health expenditure, 2012 (% of THE) from WHO [12];

Population living in endemic areas (2014) from APOC treatment database (last update:2012) and UN (population growth rates 2013–2014) [13]

SD: standard deviation

## Methods

We estimated financial costs to predict how much the governments of endemic countries and donors would have to pay for implementing the required interventions for alternative treatment goals of control, elimination, and eradication, and economic costs to assess societal opportunity costs of donated services and goods. The time horizon of the analysis was from 2013 to 2045, based on the predicted timeline for reaching the post-elimination phase in endemic African regions [9].

**Fig 1:**
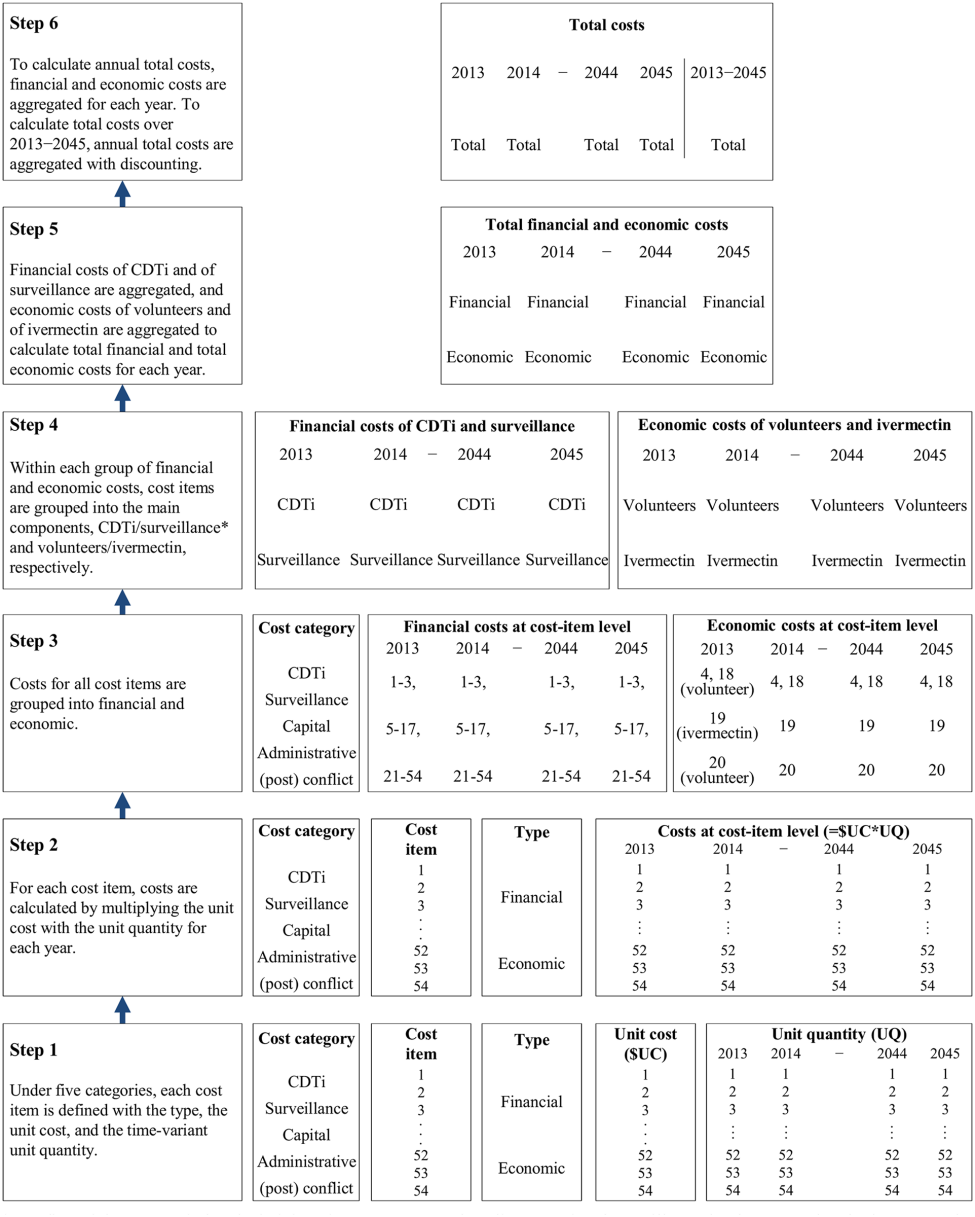
Micro-costing method for estimating total costs for a project. * For financial costs, capital and administrative costs are evenly split to CDTi and surveillance for the years when both CDTI and surveillance are implemented, and support costs for (post) conflict areas are evenly split over the entire period.

There are different methods for estimating health intervention costs, ranging from micro-costing (bottom-up approach) to gross-costing (top-down approach) [14]. We used a micro-costing method to more precisely estimate time-variant resource utilization depending on epidemiological trends and to incorporate the heterogeneity in the demographic, epidemiological, and political situation at project level. Fig 1 shows the six steps of the micro-costing approach calculating from the cost of a single cost item to the total financial and economic cost for a project.

We defined the key activities and resources required for onchocerciasis elimination and eradication with reference to an APOC report of the technical consultative committee [15], an APOC protocol for epidemiological surveillance, and a guide for post-treatment epidemiological and entomological surveillance (developed for the Onchocerciasis Elimination Program for the America) [16]. Based on the identified activities and resources, we defined cost items under five categories—CDTi, surveillance, capital costs, overhead and administrative costs, financial support for (post) conflict endemic areas—and their characteristics which include the type (financial or economic), the unit cost, and the time-variant unit quantity (depending on relevant phases among the three phases of treatment, confirmation of elimination, and post-elimination). The details about each step of the micro-costing approach and the characteristics of cost items are described in S1 Text.

### Data

We obtained 2012 budgets from APOC, approved for onchocerciasis CDTi, that cover 67 of all 112 ongoing projects (as of November 2013) in sub-Saharan Africa. All budget documents include information on the unit cost and the unit quantity of each resource, demography, available human resources (community health workers, community volunteers), and funding from the ministry of health, APOC, and non-governmental organizations. These data were used as the main sources to estimate financial costs. To estimate economic costs, agriculture value added per worker was used as an opportunity cost of community volunteers’ unpaid time [17], considering most volunteers are farmers in remote rural areas [18]. The opportunity cost of donated ivermectin was $1.5054 per treatment (three 3mg-tablets), based on Merck’s suggested drug price of $1.5 per treatment before the donation was decided [19] and on the insurance and freight cost of $0.0018 per tablet [20].

For projects with missing unit costs, we used the national average if relevant unit costs were available; otherwise, the regional average (Table 2) across available national averages for endemic African countries. For the countries that did not have agriculture value added per worker, we used the regional average for sub-Saharan Africa [17]. For projects with missing data for the determinants of unit quantities (e.g., the ratio of health workers over population, the ratio of volunteers over population), we used the national average if relevant data were available; otherwise, the regional average across available national averages for endemic African countries. Unit costs and the determinants of unit quantities at the country and regional levels are included in S1 Text.

**Table 2:** Average unit costs across endemic African countries.

Cost items	Average (SD)*	Unit
**Category 1. Community-directed treatment with ivermectin**		
**Advocacy/sensitization/mobilization**		
Advocacy	$4,544.00 ($3,624.71)	/project
Sensitization	$3,902.00 ($2,590.79)	/project
Mobilization	$0.01 ($0.01)	/person
Support for mobilization from community volunteers^%^	$1.68 ($3.12)	/volunteer/day
Development of IEC^&^ material	$2,250.00 ($1,715.51)	/project
Production of IEC^&^ material	$0.04 ($0.05)	/person
**Supervision/monitoring/evaluation**		
Supervision (first 6 years)	$18,021.00 ($16,252.27)	/project
Assistance for supervisory visits (7^th^ year+)	$2,099.00 ($981.23)	/project
Monitoring	$3,622.00 ($3,259.28)	/CDTi round
Evaluation	$4,008.00 ($599.03)	/CDTi round
Review meeting	$6,746.00 ($5,206.34)	/project
Data management	$2,309.00 ($2,287.10)	/project
Community self-monitoring	$0.02 ($0.02)	/person
**Training**		
Training of trainers and health workers	$184.00 ($334.96)	/health worker
Training of community volunteers	$8.00 ($6.31)	/volunteer
Training of community leaders	$9.00 ($7.45)	/community
**Drug distribution/management of severe adverse events**		
Community registration	$12.00 ($10.91)	/community
Census^%^	$1.68 ($3.12)	/volunteer/day
1) In areas without epidemiological challenges		
Delivery of ivermectin	$1.51 ($0.00)	/treatment
Ivermectin administration^%^	$1.68 ($3.12)	/volunteer/day
2) In areas with epidemiological challenges (co-endemicity with *Loa loa*)		
Diagnostic tools (annuitized)	$120.00 ($0.00)	/set
Delivery and administration of doxycycline^@^	$2.50 ($0.00)	/6-week treatment
Management of severe adverse events	$2,993.00 ($3,888.44)	/project
**Category 2. Surveillance**		
**Supervision/monitoring/evaluation**		
Supervisory visit	$2,099.00 ($981.23)	/project
Monitoring	$3,622.00 ($3,259.28)	/project
Evaluation	$4,008.00 ($599.03)	/project
Review meeting	$6,746.00 ($5,206.34)	/project
Data management	$2,309.00 ($2,287.10)	/project
**Training**		
Training of trainers and health workers	$184.00 ($334.96)	/health worker
Training of community volunteers (fly/larva-catchers)	$8.00 ($6.31)	/volunteer
Training of community leaders	$9.00 ($7.45)	/community
**Epidemiological survey sampling**		
Surveillance trip transportation	$21.00 ($16.11)	/person/day/site
Personnel	$15.00 ($16.72)	/person/day/site
Field supplies (annuitized)	$68.00 ($0.00)	/set
**Entomological survey sampling**		
Personnel	$2.41 ($2.14)	/person/day/site
Field supplies (annuitized)	$1.85 ($0.00)	/set/person/day/site
**Delivery of samples**		
Delivery of skin-snip samples from villages to laboratory	Included in the surveillance trip transportation costs	/site
Delivery of vector samples from catching site to health facility	$7.95 ($5.25)	/site
Delivery of vector samples from health facility to MSDC	$135.00 ($0.00)	/project
**Epidemiological laboratory testing**		
Personnel	$15.00 ($16.72)	/person/day/site
Laboratory supplies (annuitized)	$120.00 ($0.00)	/set
**Entomological laboratory testing**		
Personnel	$9.00 ($0.00)	/person/day/site
**Category 3. Capital costs**		
Vehicle (annuitized)	$3,919.00 ($661.27)	/vehicle
Motorcycle (annuitized)	$503.00 ($155.89)	/motorcycle
Bicycle (annuitized)	$21.00 ($6.12)	/bicycle
IT equipment and power supply equipment (annuitized)	$2,695.00 ($119.06)	/set
**Category 4. Overhead and administrative costs**		
Maintenance of vehicle	$280.00 ($85.71)	/vehicle
Maintenance of motorcycle	$84.00 ($25.71)	/motorcycle
Office supplies, communication, top-ups (first 6 years), others	$34,151.00 ($24,626.97)	/project
**Category 5. Financial support for CDTi and surveillance in (post) conflict endemic areas**		
Support for CDTi and surveillance in (post) conflict endemic areas#	$1,052,363.00 (NA)	/endemic African regions

^*^ Average unit costs across national averages for endemic African countries with budgets available

^%^ Agriculture value added per worker [17], 2012 GDP per capita [10]

^&^ IEC: Information/Education/Communication

^@^ Data from Wanji et al. 2009 [21]

^#^ Based on the APOC budget plan for 2008–2015 and Sightsavers’s strategic plan for 2011–2021 [22,23]

Note: all capital costs for non-disposable goods were annuitized with 3% over six years.

### Cost estimation

#### Financial costs

From an operational perspective, financial costs consist of those of CDTi and of surveillance. At project level, we multiplied the unit cost with the unit quantity for each cost item and every year. Costs of capital goods were annuitized over a useful time of each item with 3%. We assumed the useful time to be six years based on the capital-goods replacement policy specified in a Burundi’s budget document. We aggregated the costs across cost items relevant to CDTi and surveillance separately. We split the capital and administrative costs (for the years when both CDTi and surveillance were conducted) and the financial support costs for (post) conflict areas (over the entire time horizon) equally between CDTi and surveillance. To estimate annual financial costs, we added the CDTi and surveillance costs (Fig 1).

#### Economic costs

Economic costs consist of those of community volunteers, who play a central operational role in CDTi [18], and of ivermectin, the main drug for CDTi and donated by Merck. For each project, we estimated annual economic costs of community volunteers by multiplying the daily agriculture value added per worker with the number of required community volunteers (population multiplied by the ratio of volunteers over population), the required volunteering days, and the number of CDTi rounds per year. We used the multi-country survey by McFarland and colleagues [24] to identify the main activities of volunteers and the required days. Three main activities were administering ivermectin (17.8 days), supporting health workers for mobilization (5.5 days), and doing census to update treatment registers (4.6 days). As community mobilization would be required until elimination is confirmed, we included the economic costs of supporting health workers for mobilization in both phases for treatment and the confirmation of elimination.

To estimate annual economic costs of donated ivermectin, we multiplied the drug and delivery cost per treatment with the number of required treatments (population multiplied by treatment coverage and the number of CDTi rounds per year). To estimate annual economic costs for a project, we summed the annual economic costs of community volunteers and donated ivermectin (Fig 1).

#### Total costs

To estimate annual total costs, we summed annual financial and economic costs. To estimate total costs over the entire time horizon, we summed annual costs from 2013 to 2045 with discounting (Fig 1). The discount rate to account for time preference was 3%.

All costs were reported in 2012 US dollars (USD). Local currency before 2012 was inflated using country-specific inflation rates [25] and converted to USD using exchange rates [26].

### Uncertainty analysis

We conducted sensitivity analysis to assess the robustness of results to parametric uncertainties. The parameters included either cost items for which unit costs were missing for more than one third of total projects or total countries with available budgets. Also the parameters included the time-variant determinants of unit quantities: population living in endemic areas, the number of required treatments (determined by population, treatment coverage linked to required treatment duration, and possible delay in starting and ending treatments), the number of required community volunteers (determined by population and the ratio of community volunteers over population), and the number of required community health workers (determined by population and the ratio of community health workers over population). We conducted one-way sensitivity analysis to examine the impact of parameters related to CDTi performance, the cost items with high uncertainty, and discount rates on total costs. We conducted multivariate probabilistic sensitivity analysis (PSA) to examine the joint effects of uncertainties about all selected variables on total costs. For PSA, we attached statistical distributions to the selected cost items and the determinants of unit quantities, and fitted to relevant data. S1 Text describes the methodological details of the sensitivity analysis.

## Results

### Total costs

**Fig 2:**
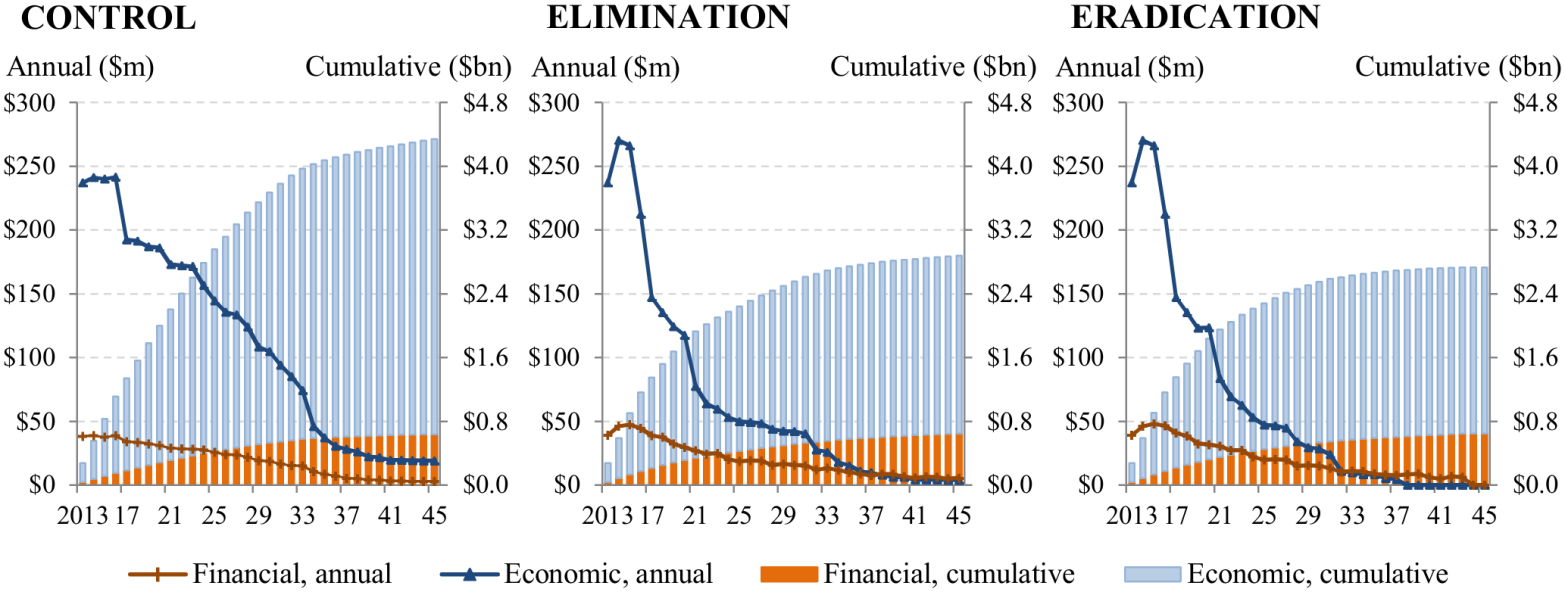
Annual and cumulative financial and economic costs over 2013–2045 for the control, elimination, and eradication scenarios.

Total financial and economic costs would be concentrated in the early stage during which treatments are scaled up to remaining endemic areas, and decrease as the treatment phase nears the end (Fig 2). In endemic African regions, total financial and economic costs over the period 2013–2045 would be $4.3 billion (95% central range from multivariate PSA: $3.9 billion[bn]–$5.0bn) for the control scenario, $2.9 billion ($2.6bn–$3.4bn) for the elimination scenario, and $2.7 billion ($2.4bn–$3.2bn) for the eradication scenario. That is, switching from control to elimination and eradication would lead to cost-savings of $1.5 billion ($1.0bn–$1.9bn) and $1.6 billion ($1.2bn–$2.1bn), respectively (S1 Fig). The eradication scenario would lead to cost-savings of $144 million (-$25 million[M]–$462M) as compared to the elimination scenario.

**Fig 3:**
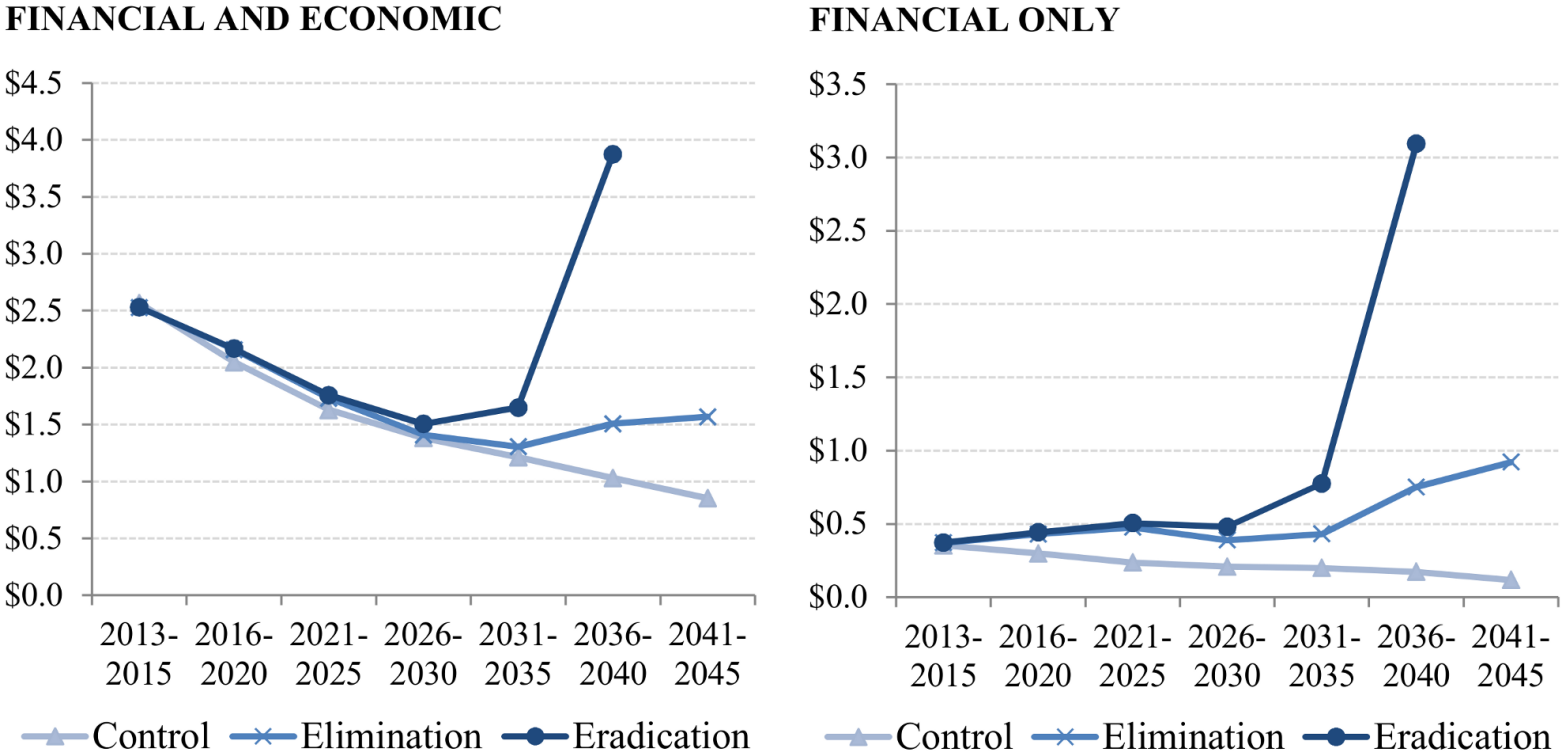
Unit costs per treatment per period for the control, elimination, and eradication scenarios, both financial and economic and only financial.

Unit financial and economic cost per treatment for the control scenario would decrease from $2.5 to $0.9 over 2013–2045. For the elimination scenario, it would decrease from $2.5 to $1.3 until 2035, and increase to $1.6 afterwards. For the eradication scenario, it would decrease from $2.5 to $1.5 over 2013–2030, and increase to $3.9 afterwards until the end of the treatment phase in endemic African regions (Fig 3).

### Financial costs

**Fig 4:**
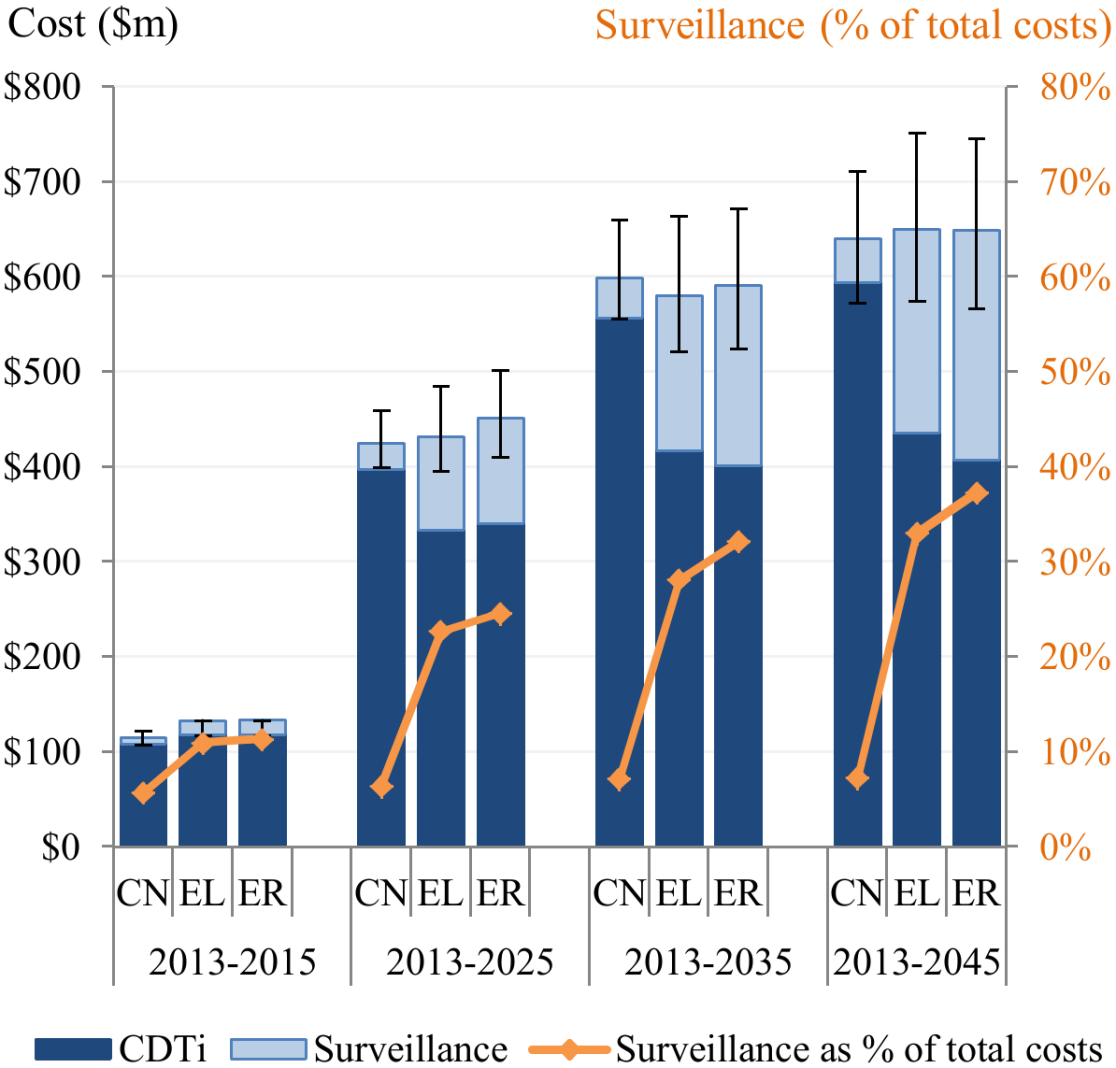
Cumulative financial costs of CDTi and surveillance over 2013–2045 for the control, elimination, and eradication scenarios. CN: control scenario, EL: elimination scenario, ER: eradication scenario

Total financial costs over the period 2013–2045 would be $640 million ($572M–$711M) for the control scenario, $650 million ($574M–$751M) for the elimination scenario, and $649 million ($566M –$745M) for the eradication scenario (Fig 4). That is, the total financial costs associated with the elimination and eradication scenarios are slightly lower than those associated with the control scenario; however, these cost differences are not robust to sensitivity analysis (S2 Fig). The main difference between scenarios is the proportion of surveillance costs in total costs. Total surveillance costs over 2013–2045 would increase from 7% ($47M) of total financial costs under the control scenario to 33% ($215M) and 37% ($242M) under the elimination and eradication scenarios, respectively (Fig 4).

Unit financial cost per treatment for the control scenario would decrease from $0.4 to $0.1 over 2013–2045. For the elimination scenario, it would stay between $0.4 and $0.5 until 2035, and increase to $0.9 afterwards. For the eradication scenario, it would stay between $0.4 and $0.5 until 2030, and increase to $3.1 as the treatment phase nears the end in endemic African regions (Fig 3).

### Economic costs

**Fig 5:**
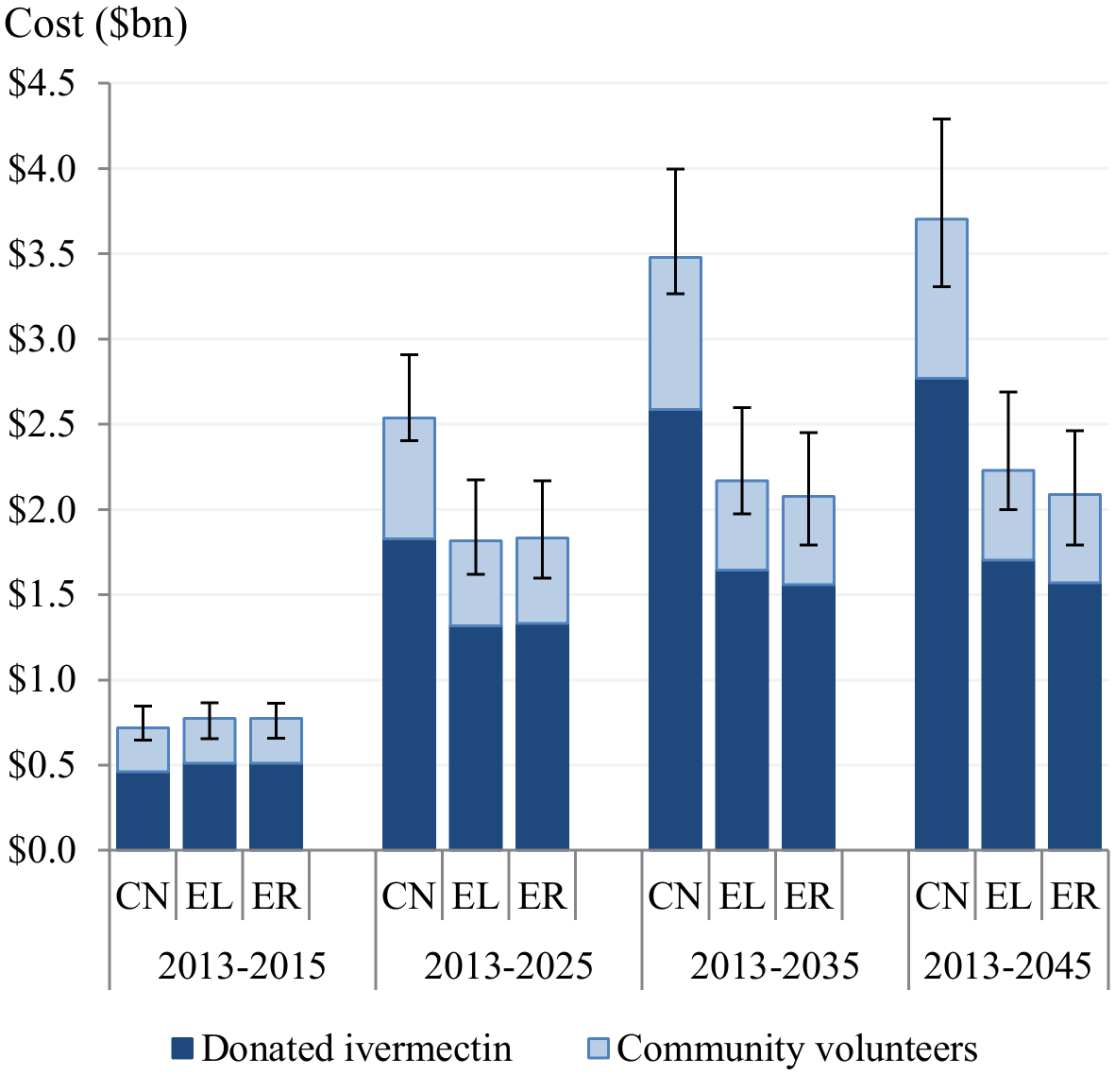
Cumulative economic costs of donated ivermectin and community volunteers’ unpaid time over 2013–2045 for the control, elimination, and eradication scenarios. CN: control scenario, EL: elimination scenario, ER: eradication scenario

Economic costs would be six times higher than financial costs under the control scenario and three times higher under the elimination and eradiation scenarios. Total economic costs over 2013–2045 would be $3.7 billion ($3.3bn–$4.3bn) for the control scenario, $2.2 billion ($2.0bn–$2.7bn) for the elimination scenario, and $2.1 billion ($1.8bn–$2.5bn) for the eradication scenario (Fig 5). That is, the total economic costs associated with the elimination and eradication scenarios are lower than those associated with the control scenario by $1.5 billion ($1.1bn–$1.9bn) and $1.6 billion ($1.2bn–$2.1bn), respectively (S3 Fig). Donated ivermectin and community volunteers would account for 75% and 25% of the total economic costs over 2013–2045 in all scenarios.

### Uncertainty analysis

**Fig 6:**
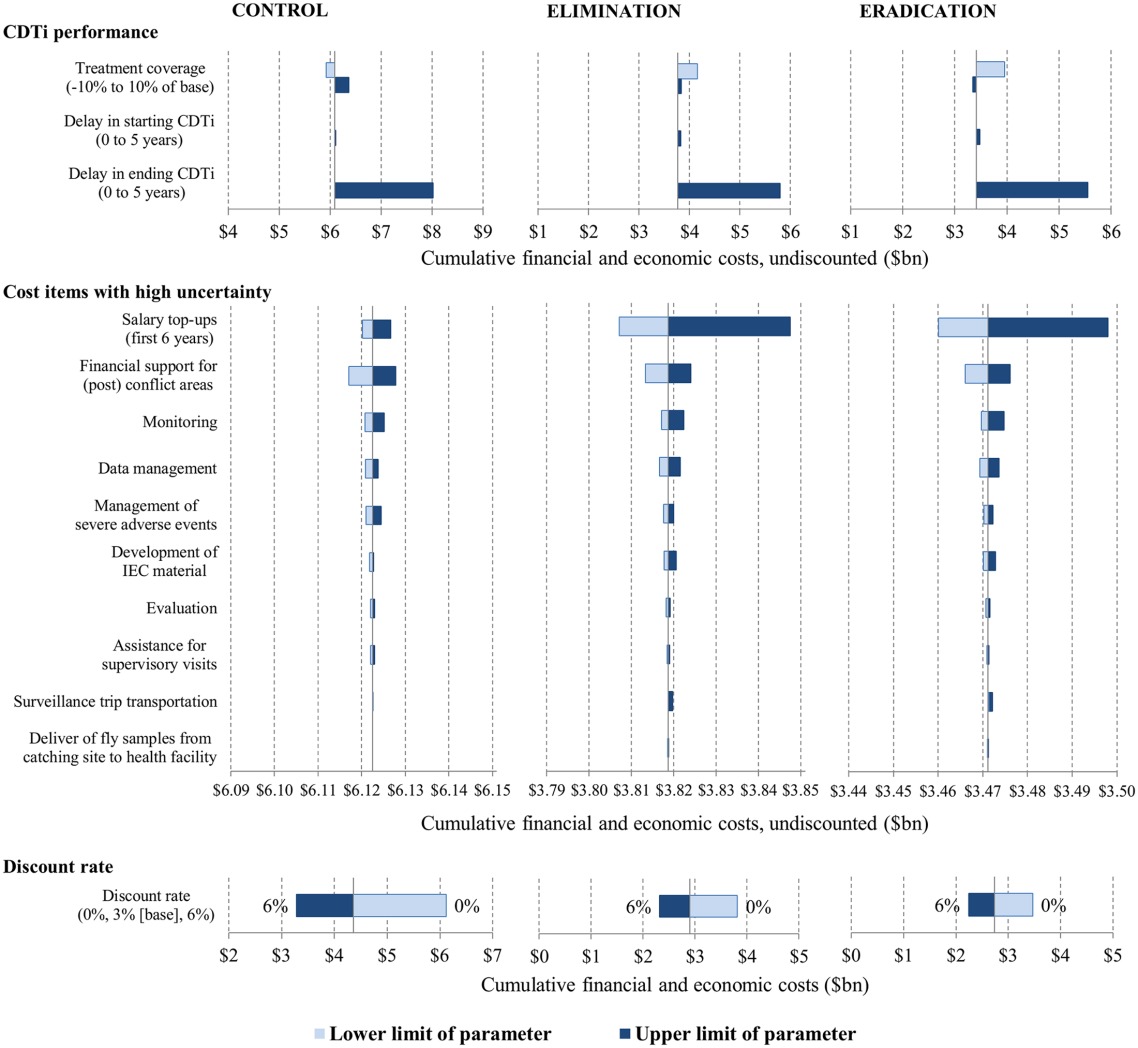
One-way sensitivity analysis for cumulative financial and economic costs over 2013–2045. One-way sensitivity analysis for CDTi performance parameters and discount rate is deterministic (using the lower and upper limits); that for cost items is probabilistic (using gamma distributions fitted to relevant data).

One-way sensitivity analysis (Fig 6) shows that, among the parameters related to CDTi performance, the delay in ending CDTi (after the infection levels reach the threshold for stopping CDTi) is the most influential parameter, leading total costs to increase by $2 billion (undiscounted) over 2013–2045 in all scenarios. Among the cost items with high uncertainty (based on the number of missing data), the most influential one is the salary top-ups for stabilizing new projects in the elimination and eradication scenarios, leading total costs (undiscounted) to range from $3.807 billion to $3.847 billion, and from $3.460 billion to $3.498 billion, respectively. Increasing the discount rate from 0% to 6% would decrease total costs over 2013–2045 by 46% from $6.1 billion to $3.3 billion for the control scenario, by 39% from $3.8 billion to $2.3 billion for the elimination scenario, and by 35% from $3.5 billion to $2.2 billion for the eradication scenario.

## Discussion

The elimination and eradication scenarios are predicted to generate substantial cost-savings in the long run compared to the control scenario. The main factors contributing to cost-savings are the reduction in economic costs of community volunteers and donated ivermectin due to a shorter treatment phase as a result of regular active surveillance. This finding implies that the saved volunteers’ time and ivermectin can be used for other health programs. Willing volunteers and their well-established networks, which have enabled successful implementation of CDTi in Africa, could contribute to improving access to primary health care in remote rural areas with insufficient human resources. In addition, the saved ivermectin drugs could be used for other disease programs, for example, anti-LF mass drug administration. To realize these possibilities, policymakers would need to keep empowering community volunteers through training and societal or economic appreciation. Also, pharmaceutical companies’ continuous commitment to donating drugs would be needed.

The main operational difference between the elimination/eradication scenarios and the control scenario is regular active surveillance. Our analysis shows that the cumulative financial costs for surveillance over 2013–2045 in the elimination and eradication scenarios would be five times higher those in the control scenario. This implies that endemic countries would need to improve their domestic funding capacity to sustain high surveillance costs to achieve elimination, as the post-treatment surveillance period could last beyond 2045 [9] and external funding would be temporary. The development and operationalization of new affordable and effective diagnostic tools, for example, OV-16 (ELISA and Rapid Test) and the DEC patch test under development [27,28], might lead to the savings of surveillance costs.

The financial unit cost per treatment in the elimination and eradication scenarios would increase by factors of respective two and eight as the regional intervention phase nears the end. This increase is driven by the reduction in the number of people in need of treatment and steady or increasing costs for surveillance and capital goods. Additionally, in the last stage, the majority of people in need of treatment are expected to live in areas with epidemiological and political challenges [9]. This implies that, in the last mile towards elimination and eradication, political, financial, and societal commitment across a whole spectrum of stakeholders will be essential to meet high unit costs and to deliver treatments in challenging areas [29]. Studies based on social choice theory and game theory [30–33] show that the elimination and eradication of infectious diseases are public goods that can only be achieved through the coordinated efforts of multiple countries. These studies suggest that high benefit-cost ratios associated with elimination and/or eradication could incentivize endemic countries to pursue elimination and/or eradication and global donors to finance endemic countries lacking the financial capacity. Equity and social justice arguments for elimination and eradication [34,35] could also complement and strengthen the economic rationality. The role of global stakeholders can play a decisive role to overcome national challenges. A study by Shaffer suggests that, to prevent potential holdout problems caused by unwilling or unable countries, which could hinder elimination and eradication, the centralized efforts led by international organizations would be necessary [36]. In line with this, it has been argued that the explicit inclusion of NTDs elimination in the Sustainable Development Goals (SDGs) of the United Nations (UN) [37,38] would further motivate the commitment of national and global policymakers and donors. Societal commitment at local level will be also essential, because delivering treatments to operationally challenging areas would require successful drug administration by community volunteers and communities’ compliance to treatments. To promote such commitment by communities, endemic countries’ continuous investments in enhancing the operational capacity of community volunteers and in mobilizing communities will be needed.

The uncertainty analysis showed that the delay in ending CDTi would have the highest impact among those related to CDTi performance on total costs. Thus, planning to move towards the post-treatment phase, along with regular monitoring and evaluation to decide the proper time of stopping treatments, would be important to avoid the delay in ending CDTi.

The uncertainty analysis also showed that the salary top-ups for stabilizing new projects would have the most influence of all cost items on total costs. Many new projects are in potential hypo-endemic areas where parasitological surveys are still needed to confirm endemicity [39]. This suggests that complete epidemiological mapping should be a priority to choose areas to start new projects and to predict required human resources for those projects.

The results presented in this study should be interpreted considering the limitations of the approach and data used. To calculate financial costs for projects without available budgets, we relied on national or regional average unit costs which might only approximately represent the actual costs in those projects. For economic costs, we assumed agriculture value added per worker as an opportunity cost of community volunteers’ unpaid time. However, other studies used different proxies such as national minimum wage and GNI per capita [24,40]. We did not use national minimum wage, as it was unavailable for 11 of 28 endemic countries [41]. We did not use GNI per capita, as it does not represent the income level in remote rural areas. In the opportunity cost of donated ivermectin, we did not include tax deduction provided to donating manufacturers [19], as the relevant detailed information is proprietary and unavailable.

There were some other factors that could affect resource utilization, but were not included in the analysis. We assumed no recrudescence, because it was difficult to predict when recrudescence would happen. If that were to happen, costs would increase because the treatment phase would have to be restarted. We did not consider the potential impact of new diagnostic and treatment tools, because it was difficult to predict when they would be developed and operationalized. If new effective and affordable tools are operationalized, the strategies of treatment and surveillance could change, thereby influencing costs. We assumed no unexpected political unrest that could interrupt interventions and would increase costs to restart the interventions.

Despite these limitations, to our knowledge and based on literature review (see S1 Text), our study is the most up-to-date cost analysis of potential regional elimination strategies in Africa. National and global policymakers and donors could use our cost analysis to make informed policy decisions and to predict the funding needs for implementing elimination programs in Africa. Our cost estimates could also be used by policymakers and researchers to compare costs and potential benefits associated with potential elimination strategies in Africa.

## Supporting Information

S1 Text Methodological details on the micro-costing method, the uncertainty analysis, and the literature review. (DOCX)

S1 Fig Incremental cumulative financial and economic costs over 2013–2045. CN: control scenario, EL: elimination scenario, ER: eradication scenario
(TIF)

S2 Fig Incremental cumulative financial costs over 2013–2045. CN: control scenario, EL: elimination scenario, ER: eradication scenario
(TIF)

S3 Fig Incremental cumulative economic costs over 2013–2045. CN: control scenario, EL: elimination scenario, ER: eradication scenario
(TIF)
