# Supplementary material for: Financial and Economic Costs of the Elimination and Eradication of Onchocerciasis (River Blindness) in Africa
Source: PLoS Negl Trop Dis. 2015 Sep 11;9(9):e0004056. doi: 10.1371/journal.pntd.0004056 (PMC4567329; doi:10.1371/journal.pntd.0004056)
Supplement: S1 Text — (DOCX) [file pntd.0004056.s001.docx]

Financial and economic costs of the elimination and eradication of onchocerciasis (river blindness) in Africa

S1 Text. Methodological details on the micro-costing method, the uncertainty analysis, and the literature review

Contents

[I. Micro-costing method 2](#_Toc427849188)

[1. Geographic unit for costing: project 2](#_Toc427849189)

[2. Cost estimation for a project 2](#_Toc427849190)

[Step 1. Identification of ingredients 2](#_Toc427849191)

[Step 2. Costs at cost-item level 13](#_Toc427849192)

[Step 3. Classification into financial and economic costs 13](#_Toc427849193)

[Step 4. Sub-classification of financial and economic costs 14](#_Toc427849194)

[Step 5. Total financial and economic costs 14](#_Toc427849195)

[Step 6. Total costs for a project 15](#_Toc427849196)

[3. Total costs for a scenario 15](#_Toc427849197)

[II. Uncertainty analysis 16](#_Toc427849198)

[1. Selection of variables 16](#_Toc427849199)

[2. Statistical distributions 16](#_Toc427849200)

[3. Simulation 20](#_Toc427849201)

[III. Literature review 21](#_Toc427849202)

# I. Micro-costing method

## 1. Geographic unit for costing: project

A unit for micro-costing was a project, considering budgets and operational decisions for community-directed treatment with ivermectin (CDTi) are made at project level in endemic African regions [1]. We used data available in Kim et al. (S1 Table in [2]) to identify the list of projects and the demographic and epidemiological characteristics of each project. The dataset included population living in endemic areas, pre-control endemicity, feasibility concerns, CDTi start year, CDTi rounds per year, expected treatment coverage, and predicted CDTi end year for the control, elimination, and eradication scenarios.

## 2. Cost estimation for a project

To estimate total costs for a project, we used a micro-costing method with six steps.

### Step 1. Identification of ingredients


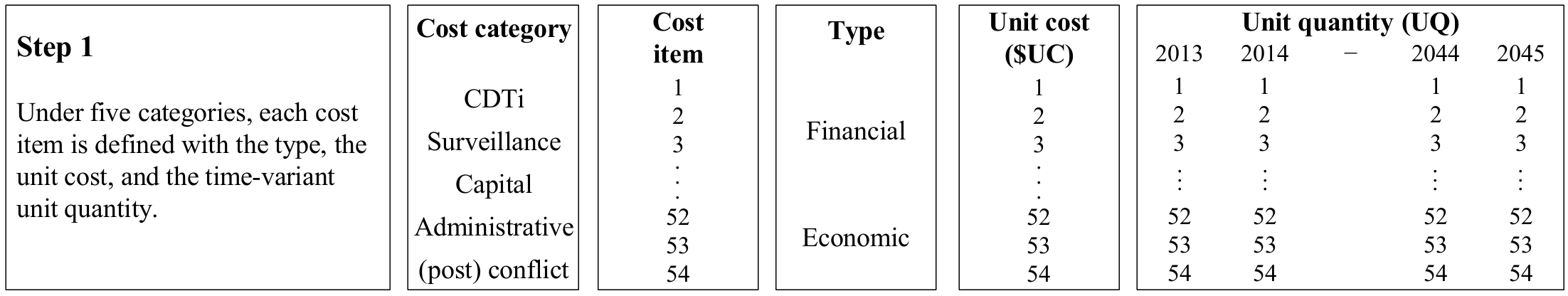


We defined the key activities and resources required for onchocerciasis elimination and eradication under five categories (S1 Table) with reference to an APOC report of the technical consultative committee [3], an APOC protocol for epidemiological surveillance, and a guide for post-treatment epidemiological and entomological surveillance (developed for the Onchocerciasis Elimination Program for the Americas) [4] . Based on the key activities and resources, we identified 54 cost items, and defined their characteristics including the type (financial/economic), the unit cost, and the unit quantity. As resource utilization, represented by the unit quantity, changes depending on epidemiological trends, we defined relevant phases for each cost item, among the phase 1 for treatment, the phase 2 for the confirmation of elimination, and the phase 3 for post-elimination surveillance (S2 Table).

**S1 Table.** **Key activities and required resources**

| **Item** | **Definition** | **Resources** |
| --- | --- | --- |
| **Category 1 Community-directed treatment with ivermctin (CDTi)** | | |
| **Advocacy/sensitization/mobilization** | | |
| Advocacy | Hold meetings at regional/province/state level to create and sustain awareness | Per diem, travel expenses, facility rent, printing |
| Sensitization and mobilization | Educate, sensitize, and mobilize communities to achieve high treatment coverage | Community volunteers, per diem, travel expenses, media announcement costs, mobilization material (town criers, banners, printing) |
| Development and production of Information/Education/Communication (IEC) material | Develop (every five years) and print (every year) training manuals and IEC material | Per diem, travel expenses, printing |
| **Supervision/monitoring/evaluation** | | |
| Supervision (first 6 years) | Supervise at district/health facility/community level to ensure smooth start of CDTi | Per diem, travel expenses |
| Assistance for supervisory visits (7^th^ year and onward) | Regular visits to districts, health facilities and communities by coordinators, epidemiologists, data managers, and accountants | Per diem, travel expenses |
| Monitoring and evaluation | Track and evaluate CDTi performance | Per diem, travel expenses |
| Review meeting | Hold regular meetings at state and district levels | Per diem, travel expenses |
| Data management | Build database, enter, and validate data | Per diem, travel expenses |
| Community self-monitoring | Track CDTi performance, report to health workers | Community volunteers, travel expenses |
| **Training** | | |
| Training of trainers and health workers | Develop capacity of trainers and health workers | Per diem, travel expenses, facility rent, printing, stationery |
| Training of community volunteers (community drug distributors) | Train community volunteers to implement CDTi | Per diem, travel expenses, printing, stationery |
| Training of community leaders | Educate community leaders on the concept of and the need for CDTi | Per diem, travel expenses, printing, stationery |
| **Drug distribution/management of severe adverse events** | | |
| Community registration | Register communities | Community volunteers, registration forms |
| Census | Conduct census | Community volunteers |
| Delivery of drugs | Deliver drugs from manufacturer to country | Drug purchase cost, shipping cost (insurance, freight) |
| Drug administration in areas without highly endemic *Loa loa* | Community-directed treatment with ivermectin | Community volunteers |
| Drug administration in areas with highly endemic *Loa loa* | A test-and-treat approach with doxycycline | Diagnostic tools for microscopic testing**^i^**, health workers |
| Management of severe adverse events | Purchase drugs, treat minor adverse events, refer people with severe adverse events to health facilities | Community volunteers, drugs |
| **Category 2 Surveillance** | | |
| **Supervision/monitoring/evaluation** | | |
| Supervisory visit | Regular visit to sentinel sites by coordinators, epidemiologists, entomologists, data managers, and accountants | Per diem, travel expenses |
| Monitoring and evaluation | Track and evaluate surveillance performance | Per diem, travel expenses |
| Review meeting | Hold regular meetings at state and district levels | Per diem, travel expenses |
| Data management | Build database, enter and validate data | Per diem, travel expenses |
| **Training** | | |
| Training of trainers and health workers | Develop capacity of trainers and health workers for epidemiological and entomological surveillance | Per diem, travel expenses, facility rent, printing, stationery |
| Training of fly/larva-catchers | Train fly/larva-catchers | Per diem, travel expenses, printing, stationery |
| Training of community leaders | Educate community leaders on the concept of and the need for surveillance | Per diem, travel expenses, printing, stationery |
| **Sampling** | | |
| Epidemiological sampling by health workers | Conduct skin snip | Per diem and travel expenses for skin-snipper, laboratory technician, and census clerk, field supplies**^ii^** |
| Entomological sampling by fly/larva-catchers | Collect aquatic (to determine catching sites) and adult stages of black flies | Perdiem for fly/larva-catchers, field supplies**^iii^** |
| **Delivery of samples** | | |
| Delivery of samples to laboratory | Carry skin-snip samples to laboratory | Travel expenses for the survey team |
| Delivery of samples to laboratory | Send fly/larva samples to MDSC(Multi Disease Surveillance Center ) in Burkina Faso**^iv^** | Transportation expenses, courier service fee |
| **Laboratory testing** | | |
| Epidemiological laboratory testing | Test skin-snip samples in laboratory | Salary for laboratory technicians, laboratory supplies**^v^** |
| Entomological laboratory testing | Test fly/larva samples | Salary for MDSC laboratory technicians**^vi^** |
| **Category 3 Capital goods^vii^** | | |
| Vehicle | Vehicle with 6 years of useful time | Vehicle |
| Motorcycle | Motorcycle with 6 years of useful time | Motorcycle |
| Bicycle | Bicycle with 6 years of useful time | Bicycle |
| IT equipment | IT equipment with 6 years of useful time | Computer, fax, printer, photocopier, scanner, projector, camera, TV, DVD reader |
| Power supply equipment | Power supply equipment with 6 years of useful time | Generator, uninterruptible power supply |
| **Category 4 Administrative costs** | | |
| Maintenance of vehicle | Maintenance of vehicles | Technician’s service fee |
| Maintenance of motorcycle | Maintenance of motorcycles | Technician’s service fee |
| Office supplies | Stationery | Stationery |
| Communication | Telephone, internet, courier | Fee for telephone, internet, and courier |
| Salary top-ups (first 6 years) | Provide salary top-ups to health workers for the first 6 years to stabilize new projects | Salary top-ups |
| Other administration | Other administrative costs | Bank charges, other administrative costs |
| **Category 5 Financial support for treatment and surveillance in (post) conflict endemic areas** | | |
| Financial support for treatment and surveillance in (post) conflict endemic areas | Strengthen infrastructure, human capacity, and monitoring and evaluation system, treat internally displaced people | Funding for the relevant activities |
| **i,v** 1ml size test tube, binocular microscope, blood slids, hypodermic syringe and needle, micro pipette with disposable tips, microtitration trays, saline solution, slide trays; the useful time of non-disposable items is assumed to be six years.  **ii** 2 liters of distilled water, 200 glass slides, 3 instrument trays, 4 slide trays each holding 3 glass slides, liquid detergent, butane burning stove, dropper bottle, aluminium pressure sterilizer, cotton swabs soaked with alcohol, curved tweezer, holth punch, lancets, scissors, sterilizer forcepts; the useful time of non-disposable items is assumed to be six years.  **iii** aspirators, bottles; the useful time of these supplies is assumed to be six years.  **iv,vi** Exceptional countries were Ethiopia, Uganda, and Sudan that were identified to conduct entomological tests in their own laboratories.  **vii** Six years of useful time is assumed following the replacement policy specified in a Burundi’s budget document | | |

**S2 Table. Characteristics of cost items: unit cost, unit quantity, relevant phase, and type**

| **ID** | **Cost item** | **Unit cost** | **Unit quantity** | **Phase^i^** | | | **Financial/ economic** |
| --- | --- | --- | --- | --- | --- | --- | --- |
|  |  |  |  | **1** | **2** | **3** |  |
| **Category 1 Community-directed treatment with ivermectin** | | | | | | | |
| **Advocacy/sensitization/mobilization** | | | | | | | |
| 1 | Advocacy | Costs for resources per project | 1 | v | v |  | Financial |
| 2 | Sensitization | Costs for resources per project | 1 | v | v |  | Financial |
| 3 | Mobilization | Costs for resources per person | Population in a project area | v | v |  | Financial |
| 4 | Support for mobilization from community volunteers | Opportunity cost per volunteer-day | 5.5days[5]*No. of volunteers*CDTi rounds/year | v | v |  | Economic |
| 5 | Development of IEC material | Costs for resources per project | 1 | v | v |  | Financial |
| 6 | Production of IEC material | Costs for resources per person | Population in a project area | v | v |  | Financial |
| **Supervision/monitoring/evaluation** | | | | | | | |
| 7 | Supervision (first 6 years) | Costs for resources per project | 1 | v |  |  | Financial |
| 8 | Assistance for supervisory visits (7^th^ year+) | Costs for resources per project | 1 | v | v |  | Financial |
| 9 | Monitoring | Costs for resources per CDTi round | Number of CDTi rounds per year | v | v |  | Financial |
| 10 | Evaluation | Costs for resources per CDTi round | Number of CDTi rounds per year | v | v |  | Financial |
| 11 | Review meeting | Costs for resources per project | 1 | v | v |  | Financial |
| 12 | Data management | Costs for resources per project | 1 | v | v |  | Financial |
| 13 | Community self-monitoring | Costs for resources per person | Population in a project area | v | v |  | Financial |
| **Training** | | | | | | | |
| 14 | Training of trainers and health workers | Costs for resources per health worker | No. of health workers | v |  |  | Financial |
| 15 | Training of community volunteers | Costs for resources per volunteer | No. of volunteers | v |  |  | Financial |
| 16 | Training of community leaders | Costs for resources per community | No. of communities | v |  |  | Financial |
| **Drug distribution** | | | | | | | |
| 17 | Community registration | Costs for resources per community | No. of communities | v |  |  | Financial |
| 18 | Census | Opportunity cost per volunteer-day | 4.6days[5]*No. of volunteers*CDTi rounds/year | v |  |  | Economic |
|  | **Drug delivery and administration in areas without highly endemic *Loa loa* (19, 20)** | | | | | | |
| 19 | Delivery of ivermectin | Opportunity cost per treatment | No. of treatments | v |  |  | Economic |
| 20 | Ivermectin administration | Opportunity cost per volunteer-day | 17.8days[5]* No. of volunteers*CDTi rounds/year | v |  |  | Economic |
|  | **Drug delivery and administration in areas with highly endemic *Loa loa* (21, 22)** | | | | | | |
| 21 | Diagnostic tools | Purchase cost per set of diagnostic tools (annuitized) | 1 | v |  |  | Financial |
| 22 | Delivery and administration of doxycycline | Costs per 6-week treatment | No. of treatments | v |  |  | Financial |
| 23 | Management of severe adverse events | Costs for resources per project | 1 | v |  |  | Financial |
| **Category 2 Surveillance^ii^** | | | | | | | |
| **Supervision/monitoring/evaluation** | | | | | | | |
| 24 | Supervisory visit | Costs for resources per project | 1 | v | v | v | Financial |
| 25 | Monitoring | Costs for resources per project | 1 | v | v | v | Financial |
| 26 | Evaluation | Costs for resources per project | 1 | v | v | v | Financial |
| 27 | Review meeting | Costs for resources per project | 1 | v | v | v | Financial |
| 28 | Data management | Costs for resources per project | 1 | v | v | v | Financial |
| **Training** | | | | | | | |
| 29 | Training of trainers and health workers | Costs for resources per health worker | No. of health workers | v | v | v | Financial |
| 30 | Training of fly/larva catchers | Costs for resources per flycatcher | No. of fly/larva -catchers (4 per catching site)*No. of catching sites |  | v | v | Financial |
| 31 | Training of community leaders | Costs for resources per community | No. of communities | v | v | v | Financial |
| **Sampling and laboratory testing** | | | | | | | |
|  | **Epidemiological survey sampling** | | | | | | |
| 32 | Surveillance trip transportation | Transportation costs per person-day per site | No. of survey workers(3)*2days*No. of survey sites | v | v | v | Financial |
| 33 | Personnel | Personnel costs per person-day per site | No. of survey workers(3)*2days*No. of survey sites | v | v | v | Financial |
| 34 | Field supplies | Purchase cost per set of supplies (annuitized) | 1 | v | v | v | Financial |
|  | **Entomological survey sampling** | | | | | | |
| 35 | Personnel | Personnel costs per person-day per site | No. of fly/larva catchers(4)*16days (4 days/month and 4 months)*No. of catching sites |  | v | v | Financial |
| 36 | Field supplies | Purchase cost per set of supplies per person-day (annuitized) | No. of fly/larva catchers(4)*16days (4 days/month and 4 months)*No. of catching sites |  | v | v | Financial |
|  | **Delivery of samples** | | | | | | |
| 37 | Delivery of samples from villages to laboratory | Transportation costs per site | No. of survey sites | v | v | v | Financial |
| 38 | Delivery of samples from catching site to health facility | Transportation costs per site | No. of fly/larva catchers(4)*4 (1 delivery/month and 4 months)*No. of catching sites |  | v | v | Financial |
| 39 | Delivery of samples from health facility to MSDC | Courier fee per parcel | 1 |  | v | v | Financial |
|  | **Epidemiological laboratory testing** | | | | | | |
| 40 | Personnel | Personnel costs per person-day per site | No. of technicians (1)* 2days*No. of survey sites | v | v | v | Financial |
| 41 | Laboratory supplies | Purchase cost per set of supplies (annuitized) | 1 | v | v | v | Financial |
|  | **Entomological laboratory testing** | | | | | | |
| 42 | Personnel | Personnel costs per technician-day per site | No. of technicians (6)**^iii^***22days for testing*No. of catching sites |  | v | v | Financial |
| **Category 3 Capital goods^iv^** | | | | | | | |
| 43 | Vehicle | Purchase cost per vehicle (annuitized) | 1 | v | v | v | Financial |
| 44 | Motorcycle | Purchase cost per motorcycle (annuitized) | No. of districts | v | v | v | Financial |
| 45 | Bicycle | Purchase cost per bicycle (annuitized) | No. of communities | v | v | v | Financial |
| 46 | IT equipment | Purchase cost per set of equipment (annuitized) | 1 | v | v | v | Financial |
| 47 | Power supply equipment | Purchase cost per set of equipment (annuitized) | 1 | v | v | v | Financial |
| **Category 4 Administrative costs** | | | | | | | |
| 48 | Maintenance of vehicle | Maintenance costs per vehicle | No. of vehicles | v | v | v | Financial |
| 49 | Maintenance of motorcycle | Maintenance costs per motorcycle | No. of motorcycles | v | v | v | Financial |
| 50 | Office supplies | Costs for resources per project | 1 | v | v | v | Financial |
| 51 | Communication | Costs for resources per project | 1 | v | v | v | Financial |
| 52 | Salary top-ups (first 6 years) | Costs for resources per project | 1 | v |  |  | Financial |
| 53 | Other administration | Costs for resources per project | 1 | v | v | v | Financial |
| **Category 5 Financial support for MDA and surveillance in (post) conflict endemic areas** | | | | | | | |
| 54 | Support for CDTi and surveillance in (post) conflict endemic areas | Costs for resources for entire (post) conflict areas | 1 | v | v | v | Financial |
| i Phase 1: intervention; Phase 2: confirmation of elimination; Phase 3: post-elimination  ii We assumed that supervisory visit, monitoring, evaluation, review meeting, data management, and training (ID:24-31) would be done only in the years when either epidemiological or entomological surveillance is conducted.  iii 5 technicians per month = 10,000 flies per catching site/100 flies per technician-day/22 working days per month; 1 additional technician per month for larva-testing to identify species.  iv Capital costs for diagnostic and laboratory testing tools are excluded; instead included in CDTi and surveillance cost items. | | | | | | | |

As the main sources to estimate unit costs, we used approved budgets for 67 of ongoing 112 onchocerciasis projects (as of November 2013) in sub-Saharan Africa, which were made available by APOC. S3 Table shows the summary of unit costs at country and regional levels. The capital costs were annuitized with 3% over the useful time that was assumed to be six years based on the capital-goods replacement policy specified in a Burundi’s budget documents. For projects without available budgets, we used the national average unit costs or, if there was no national average, the regional average across available national averages for endemic African countries (S3 Table). For economic unit costs, we used agriculture value added per worker as an opportunity cost of community volunteers’ unpaid time, considering most of volunteers are farmers in remote rural areas. For three countries for which agriculture value added was unavailable, we used the regional average for sub-Saharan Africa (developing only) (S4 Table). As an opportunity cost of donated ivermectin, we used $1.5054 per treatment based on the suggested drug price ($1.5 per treatment) by Merck before their ivermectin donation was decided [6] and the freight and insurance cost ($0.0054 per treatment) [7]. To estimate the unit quantity for each cost item, we identified the determinants of unit quantities. The determinants were population living in a project area, the number of CDTi rounds per year, the number of treatments, the number of community volunteers, the number of volunteering days, the number of community health workers, the number of districts, the number of communities, the number of survey sites, the number of survey team members, the number of survey days, the number of fly-catching sites, the number of fly-catchers, the number of catching days, and the number of laboratory technicians (S2 Table). Among these, time variant determinants were population living in a project area, the number of treatments, the number of community volunteers, and the number of community health workers. To adjust for time variation, we adjusted population living in a project area for population growth rates over 2013−2045[8]. We estimated the number of treatments by multiplying the population adjusted for growth rates with the expected treatment coverage and the CDTi rounds per year. To estimate the number of community volunteers for CDTi, we multiplied the population adjusted for population growth rates with the ratio of community volunteers for the years of the treatment phase. To estimate the number of community health workers, we multiplied the population adjusted for population growth rates with the ratio of community health workers for the treatment phase and, in the post-treatment phase, for the years when surveillance is conducted. Projects without budgets available had no information on the ratio of community volunteers over population, the ratio of community health workers over population, the population per district, and the population per community. For these projects, we used the national averages or, if there was no national average, the regional average across available national averages for endemic African countries (S5 Table).

**S3 Table. Summary of unit costs at country and regional levels, average (standard deviation)**

| **ID** | **Cost items** | **Unit** | **Angola** | **Burundi** | **Cameroon** | **Central African Republic** | **Chad** | **Congo** | **Eq. Guinea^+^** | **Ethiopia** | **Liberia** | **Malawi** | **Nigeria** | **South Sudan** | **Tanzania** | **Uganda** | **Average^*^** |
| --- | --- | --- | --- | --- | --- | --- | --- | --- | --- | --- | --- | --- | --- | --- | --- | --- | --- |
|  | Number of projects with budgets/total (as of November 2013) | | 7/8 | 3/3 | 15/15 | 1/1**^#^** | 1/1**^%^** | 1/1 | 1/1 | 2/9 | 3/3 | 2/2**^@^** | 18/28 | 5/5 | 3/7 | 5/5 |  |
| **Category 1. Community-directed treatment with ivermectin** | | | | | | | | | | | | | | | | | |
| **Advocacy/sensitization/mobilization** | | | | | | | | | | | | | | | | | |
| 1 | Advocacy | /project | $7,312 ($4,788) | $4,611 ($4,840) | $6,930 ($5,371) | NA | $5,059 ($1,533) | $6,328 ($0) | $1,700 ($0) | $1,097 ($234) | $2,638 ($1,525) | $259 ($192) | $4,037 ($3,895) | NA | $13,216 ($1,505) | $1,336 ($1,231) | $4,544 ($3,624) |
| 2 | Sensitization | /project | $7,772 ($5,466) | $6,436 ($4,619) | $5,143 ($6,267) | $677 ($0) | $4,368 ($2,556) | $8,688 ($0) | $1,440 ($0) | $2,520 ($1,624) | $4,566 ($2,985) | $672 ($237) | $5,088 ($4,480) | $3,356 ($1,010) | $3,094 ($786) | $805 ($475) | $3,902 ($2,591) |
| 3 | Mobilization | /person | $0.016 ($0.019) | $0.007 ($0.005) | $0.014 ($0.024) | $0.010 ($0.010) | $0.046 ($0.027) | $0.002 ($0.000) | NA | $0.010 ($0.002) | $0.008 ($0.009) | $0.001 (>$0.000) | $0.006 ($0.005) | $0.010 ($0.006) | $0.035 ($0.011) | $0.003 ($0.001) | $0.013 ($0.013) |
| 4 | Support for mobilization from community volunteers**^a^** | /volunteer/day | $2.105 (NA) | $0.390 (NA) | $0.921 (NA) | $0.998 (NA) | $0.570 (NA) | $0.409 (NA) | $12.327 (NA) | $0.869 (NA) | $0.616 (NA) | $0.294 (NA) | $2.317 (NA) | $0.536 (NA) | $0.643 (NA) | $0.548 (NA) | $1.682 ($3.124) |
| 5 | Development of IEC material | /project | NA | NA | NA | NA | NA | NA | NA | $2,200 ($1,697) | $4,532 ($99) | NA | $1,889 ($1,566) | NA | NA | $382 ($0) | $2,250 ($1,716) |
| 6 | Production of IEC material | /person | $0.177 ($0.241) | NA | $0.043 ($0.049) | NA | NA | $0.015 ($0.000) | NA | $0.009 ($0.003) | $0.028 ($0.020) | $0.039 ($0.048) | $0.008 ($0.007) | $0.023 ($0.009) | NA | $0.006 ($0.004) | $0.039 ($0.053) |
| **Supervision/monitoring/evaluation** | | | | | | | | | | | | | | | | | |
| 7 | Supervision (first 6 years) | /project | $11,269 ($10,754) | $2,945 ($2,406) | $36,398 ($42,103) | $1,444 ($1,178) | $23,152 ($1,972) | NA | NA | $42,949 ($24,934) | $7,074 ($3,407) | $7,822 ($9,155) | $8,367 ($8,325) | $10,930 ($7,595) | $49,520 ($15,981) | $14,384 ($0) | $18,021 ($16,253) |
| 8 | Assistance for supervisory visits (7^th^ year+) | /project | $4,000 ($1,414) | $3,001 ($2,434) | $2,460 ($1,098) | NA | $635 ($352) | $2,489 ($0) | $1,450 ($0) | NA | NA | $1,253 ($655) | $1,702 ($1,480) | $1,500 ($0) | NA | $2,498 ($566) | $2,099 ($981) |
| 9 | Monitoring | /CDTi round | $5,203 ($4,630) | $1,617 ($19) | $10,482 ($7,449) | NA | $1,468 ($676) | NA | NA | $375 ($177) | $1,735 ($17) | $4,963 ($5,201) | $3,135 ($4,353) | NA | NA | NA | $3,622 ($3,259) |
| 10 | Evaluation | /CDTi round | NA | $3,585 ($0) | NA | NA | NA | NA | NA | NA | NA | $4,432 ($4,309) | NA | NA | NA | NA | $4,008 ($599) |
| 11 | Review meeting | /project | $5,293 ($4,031) | $4,037 ($0) | $16,796 ($24,483) | NA | $7,787 ($3,049) | NA | $1,419 ($0) | $13,600 ($936) | $2,573 ($889) | $3,986 ($3,978) | $7,375 ($6,721) | $3,488 ($561) | $13,409 ($1,214) | $1,191 ($0) | $6,746 ($5,206) |
| 12 | Data management | /project | $1,400 ($0) | $6,793 ($9,598) | $5,574 ($7,273) | NA | $175 ($207) | $1,463 ($0) | $800 ($0) | NA | NA | $1,344 ($540) | $975 ($2,220) | NA | NA | $2,258 ($2,210) | $2,309 ($2,287) |
| 13 | Community self-monitoring | /person | $0.049 ($0.060) | $0.004 ($0.002) | $0.014 ($0.006) | NA | $0.066 ($0.019) | $0.002 ($0.000) | NA | $0.025 ($0.018) | $0.002 ($0.001) | $0.006 ($0.005) | $0.006 ($0.006) | $0.005 ($0.002) | NA | $0.004 ($0.004) | $0.017 ($0.022) |
| **Training** | | | | | | | | | | | | | | | | | |
| 14 | Training of trainers and health workers | /health worker | $132 ($57) | $47 ($31) | $140 ($123) | $53 ($0) | $81 ($0) | $16 ($0) | NA | $1,179 ($275) | $48 ($19) | $1 ($0) | $8 ($6) | $107 ($24) | $579 ($109) | $6 ($1) | $184 ($335) |
| 15 | Training of community volunteers | /volunteer | $20 ($7) | $3 ($1) | $9 ($7) | NA | $11 ($8) | $2 ($0) | $5 ($0) | $18 ($1) | $4 ($2) | $2 ($2) | $3 ($3) | $8 ($5) | $13 ($6) | $1 ($2) | $8 ($6) |
| 16 | Training of community leaders | /community | $15 ($13) | $18 ($5) | $9 ($5) | NA | $5 ($0) | $12 ($0) | NA | $23 ($17) | NA | $0.350 ($0.003) | $5 ($6) | $4 ($3) | NA | $2 ($1) | $9 ($7) |
| **Drug distribution** | | | | | | | | | | | | | | | | | |
| 17 | Community registration | /community | $11 ($11) | $37 ($0) | $14 ($11) | $4 ($7) | $8 ($2) | $1 ($0) | $15 ($0) | $12 ($1) | $3 ($1) | $4 ($5) | $9 ($28) | $34 ($15) | $10 ($5) | $2 ($0) | $12 ($11) |
| 18 | Census**^b^** | /volunteer/day | $2.105 (NA) | $0.390 (NA) | $0.921 (NA) | $0.998 (NA) | $0.570 (NA) | $0.409 (NA) | $12.327 (NA) | $0.869 (NA) | $0.616 (NA) | $0.294 (NA) | $2.317 (NA) | $0.536 (NA) | $0.643 (NA) | $0.548 (NA) | $1.682 ($3.124) |
|  | **Drug delivery and administration in areas without epidemiological challenges (19, 20)** | | | | | | | | | | | | | | | | |
| 19 | Delivery of ivermectin | /treatment | $1.505 (NA) | $1.505 (NA) | $1.505 (NA) | $1.505 (NA) | $1.505 (NA) | $1.505 (NA) | $1.505 (NA) | $1.505 (NA) | $1.505 (NA) | $1.505 (NA) | $1.505 (NA) | $1.505 (NA) | $1.505 (NA) | $1.505 (NA) | $1.505 ($0.000) |
| 20 | Ivermectin administration**^c^** | /volunteer/day | $2.105 (NA) | $0.390 (NA) | $0.921 (NA) | $0.998 (NA) | $0.570 (NA) | $0.409 (NA) | $12.327 (NA) | $0.869 (NA) | $0.616 (NA) | $0.294 (NA) | $2.317 (NA) | $0.536 (NA) | $0.643 (NA) | $0.548 (NA) | $1.682 ($3.124) |
|  | **Drug delivery and administration in areas with epidemiological challenges^d^ (21, 22)** | | | | | | | | | | | | | | | | |
| 21 | Diagnostic tools (annuitized) | /set | $120 (NA) | $120 (NA) | $120 (NA) | $120 (NA) | $120 (NA) | $120 (NA) | $120 (NA) | $120 (NA) | $120 (NA) | $120 (NA) | $120 (NA) | $120 (NA) | $120 (NA) | $120 (NA) | $120 ($0) |
| 22 | Delivery and administration of doxycycline | /6-week treatment | $2.500 (NA) | $2.500 (NA) | $2.500 (NA) | $2.500 (NA) | $2.500 (NA) | $2.500 (NA) | $2.500 (NA) | $2.500 (NA) | $2.500 (NA) | $2.500 (NA) | $2.500 (NA) | $2.500 (NA) | $2.500 (NA) | $2.500 (NA) | $2.500 ($0.000) |
| 23 | Management of severe adverse events | /project | $1,360 ($381) | $1,070 ($840) | $5,329 ($8,271) | NA | NA | $175 ($0) | $1,275 ($0) | $2,465 ($0) | $13,195 ($4,185) | NA | $3,245 ($2,266) | $544 ($345) | NA | $1,277 ($0) | $2,993 ($3,888) |
| **Category 2. Surveillance** | | | | | | | | | | | | | | | | | |
| **Supervision/monitoring/evaluation** | | | | | | | | | | | | | | | | | |
| 24 | Supervisory visit | /project | $4,000 ($1,414) | $3,001 ($2,434) | $2,460 ($1,098) | NA | $635 ($352) | $2,489 ($0) | $1,450 ($0) | NA | NA | $1,253 ($655) | $1,702 ($1,480) | $1,500 ($0) | NA | $2,498 ($566) | $2,099 ($981) |
| 25 | Monitoring | /project | $5,203 ($4,630) | $1,617 ($19) | $10,482 ($7,449) | NA | $1,468 ($676) | NA | NA | $375 ($177) | $1,735 ($17) | $4,963 ($5,201) | $3,135 ($4,353) | NA | NA | NA | $3,622 ($3,259) |
| 26 | Evaluation | /project | NA | $3,585 ($0) | NA | NA | NA | NA | NA | NA | NA | $4,432 ($4,309) | NA | NA | NA | NA | $4,008 ($599) |
| 27 | Review meeting | /project | $5,293 ($4,031) | $4,037 ($0) | $16,796 ($24,483) | NA | $7,787 ($3,049) | NA | $1,419 ($0) | $13,600 ($936) | $2,573 ($889) | $3,986 ($3,978) | $7,375 ($6,721) | $3,488 ($561) | $13,409 ($1,214) | $1,191 ($0) | $6,746 ($5,206) |
| 28 | Data management | /project | $1,400 ($0) | $6,793 ($9,598) | $5,574 ($7,273) | NA | $175 ($207) | $1,463 ($0) | $800 ($0) | NA | NA | $1,344 ($540) | $975 ($2,220) | NA | NA | $2,258 ($2,210) | $2,309 ($2,287) |
| **Training** | | | | | | | | | | | | | | | | | |
| 29 | Training of trainers and health workers | /health worker | $132 ($57) | $47 ($31) | $140 ($123) | $53 ($0) | $81 ($0) | $16 ($0) | NA | $1,179 ($275) | $48 ($19) | $1 ($0) | $8 ($6) | $107 ($24) | $579 ($109) | $6 ($1) | $184 ($335) |
| 30 | Training of fly-catchers**e** | /fly-catcher | $20 ($7) | $3 ($1) | $9 ($7) | NA | $11 ($8) | $2 ($0) | $5 ($0) | $18 ($1) | $4 ($2) | $2 ($2) | $3 ($3) | $8 ($5) | $13 ($6) | $1 ($2) | $8 ($6) |
| 31 | Training of community leaders | /community | $15 ($13) | $18 ($5) | $9 ($5) | NA | $5 ($0) | $12 ($0) | NA | $23 ($17) | NA | $0.350 ($0.003) | $5 ($6) | $4 ($3) | NA | $2 ($1) | $9 ($7) |
| **Sampling and laboratory testing** | | | | | | | | | | | | | | | | | |
|  | **Epidemiological survey sampling** | | | | | | | | | | | | | | | | |
| 32 | Surveillance trip transportation**f** | /person/day/site | $52 ($109) | $14 ($18) | $32 ($56) | $9 ($2) | $6 ($0) | NA | NA | $7 ($1) | $21 ($3) | NA | $30 ($44) | NA | NA | NA | $21 ($16) |
| 33 | Personnel**g** | /person/day/site | $64 ($25) | $10 ($6) | $25 ($34) | $6 ($0) | $25 ($0) | $1 ($0) | $1 ($0) | $13 ($3) | $3 ($2) | $4 ($2) | $17 ($15) | $19 ($17) | NA | $8 ($10) | $15 ($17) |
| 34 | Field supplies (annuitized)**h** | /set | $68 (NA) | $68 (NA) | $68 (NA) | $68 (NA) | $68 (NA) | $68 (NA) | $68 (NA) | $68 (NA) | $68 (NA) | $68 (NA) | $68 (NA) | $68 (NA) | $68 (NA) | $68 (NA) | $68 ($0) |
|  | **Entomological survey sampling** | | | | | | | | | | | | | | | | |
| 35 | Personnel**i** | /person/day/site | $7.929 ($4.046) | $1.449 ($0.503) | $3.087 ($1.431) | NA | $1.148 ($0.000) | NA | NA | $2.500 ($0.707) | $1.000 ($0.000) | $1.468 ($0.000) | $1.559 ($1.092) | $3.330 ($2.057) | NA | $0.604 ($0.400) | $2.407 ($2.138) |
| 36 | Field supplies (annuitized) **j** | /set/person/day/site | $1.850 (NA) | $1.850 (NA) | $1.850 (NA) | $1.850 (NA) | $1.850 (NA) | $1.850 (NA) | $1.850 (NA) | $1.850 (NA) | $1.850 (NA) | $1.850 (NA) | $1.850 (NA) | $1.850 (NA) | $1.850 (NA) | $1.850 (NA) | $1.850 ($0.000) |
|  | **Delivery of samples** | | | | | | | | | | | | | | | | |
| 37 | Delivery of samples from villages to laboratory | /site | Included in the surveillance trip transportation costs (ID:32) | | | | | | | | | | | | | | |
| 38 | Delivery of samples from catching site to health facility**k** | /site | $4.670 ($3.423) | $1.127 ($0.000) | $6.145 ($7.657) | $11.740 ($8.784) | $6.250 ($0.000) | NA | NA | $12.500 ($3.536) | $5.023 ($5.096) | $18.367 ($14.874) | $5.761 ($6.033) | NA | NA | NA | $7.954 ($5.249) |
| 39 | Delivery of samples from health facility to MSDC**l** | /project | $135 (NA) | $135 (NA) | $135 (NA) | $135 (NA) | $135 (NA) | $135 (NA) | $135 (NA) | $135 (NA) | $135 (NA) | $135 (NA) | $135 (NA) | $135 (NA) | $135 (NA) | $135 (NA) | $135 ($0) |
|  | **Epidemiological laboratory testing** | | | | | | | | | | | | | | | | |
| 40 | Personnel**m** | /person/day/site | $64 ($25) | $10 ($6) | $25 ($34) | $6 ($0) | $25 ($0) | $1 ($0) | $1 ($0) | $13 ($3) | $3 ($2) | $4 ($2) | $17 ($15) | $19 ($17) | NA | $8 ($10) | $15 ($17) |
| 41 | Laboratory supplies (annuitized)**n** | /set | $120 (NA) | $120 (NA) | $120 (NA) | $120 (NA) | $120 (NA) | $120 (NA) | $120 (NA) | $120 (NA) | $120 (NA) | $120 (NA) | $120 (NA) | $120 (NA) | $120 (NA) | $120 (NA) | $120 ($0) |
|  | **Entomological laboratory testing** | | | | | | | | | | | | | | | | |
| 42 | Personnel**o** | /person/day/site | $9 (NA) | $9 (NA) | $9 (NA) | $9 (NA) | $9 (NA) | $9 (NA) | $9 (NA) | $13 ($3) | $9 (NA) | $9 (NA) | $9 (NA) | $9 (NA) | $9 (NA) | $8 ($10) | $9 ($0) |
| **Category 3. Capital costs** | | | | | | | | | | | | | | | | | |
| 43 | Vehicle (annuitized) | /vehicle | $2,931 (NA) | $5,663 (NA) | $4,103 (NA) | NA | NA | $3,538 (NA) | $3,751 (NA) | $3,918 (NA) | $3,751 (NA) | NA | $3,681 (NA) | $3,751 (NA) | $3,918 (NA) | $4,103 (NA) | $3,919 ($661) |
| 44 | Motorcycle (annuitized) | /motorcycle | $469 (NA) | $708 (NA) | $528 (NA) | NA | NA | $352 (NA) | $493 (NA) | $493 (NA) | $493 (NA) | NA | $345 (NA) | $879 (NA) | $493 (NA) | $399 (NA) | $503 ($156) |
| 45 | Bicycle (annuitized) | /bicycle | $17 (NA) | $19 (NA) | $23 (NA) | NA | NA | $22 (NA) | $22 (NA) | $22 (NA) | $15 (NA) | $ (NA) | $29 (NA) | $29 (NA) | $22 (NA) | $7 (NA) | $21 ($6) |
| 46 | IT equipment (annuitized) | /set | $2,287 (NA) | $2,330 (NA) | $2,288 (NA) | $2,171 (NA) | $2,181 (NA) | $2,181 (NA) | $2,197 (NA) | $2,181 (NA) | $2,187 (NA) | $1,924 (NA) | $2,299 (NA) | $2,132 (NA) | $2,181 (NA) | $1,998 (NA) | $2,181 ($111) |
| 47 | Power supply equipment (annuitized) | /set | $514 (NA) | $514 (NA) | $543 (NA) | $514 (NA) | $514 (NA) | $514 (NA) | $514 (NA) | $514 (NA) | $502 (NA) | $514 (NA) | $528 (NA) | $514 (NA) | $514 (NA) | $483 (NA) | $514 ($13) |
| **Category 4. Overhead and administrative costs** | | | | | | | | | | | | | | | | | |
| 48 | Maintenance of vehicle | /vehicle | $444 (NA) | $221 (NA) | $243 (NA) | $273 (NA) | $293 (NA) | $352 (NA) | $393 (NA) | $204 (NA) | $318 (NA) | $147 (NA) | $289 (NA) | $356 (NA) | $188 (NA) | $200 (NA) | $280 ($86) |
| 49 | Maintenance of motorcycle | /motorcycle | $133 (NA) | $66 (NA) | $73 (NA) | $82 (NA) | $88 (NA) | $106 (NA) | $118 (NA) | $61 (NA) | $95 (NA) | $44 (NA) | $87 (NA) | $107 (NA) | $56 (NA) | $60 (NA) | $84 ($26) |
| 50 | Office supplies | /project | $923 ($327) | $1,375 ($455) | $1,714 ($1,058) | NA | $9,605 ($0) | $4,367 ($0) | NA | $1,050 ($495) | $2,343 ($427) | $4,103 ($0) | $2,811 ($6,545) | $1,230 ($464) | $1,685 ($441) | $1,541 ($806) | $2,519 ($2,459) |
| 51 | Communication | /project | $1,944 ($1,264) | $1,739 ($256) | $4,585 ($4,136) | NA | $869 ($0) | $3,565 ($0) | NA | $1,800 ($0) | NA | NA | $1,260 ($1,178) | $1,936 ($327) | $11,973 ($2,140) | $745 ($752) | $3,042 ($3,354) |
| 52 | Salary top-ups (first 6 years) | /project | $14,708 ($8,284) | $18,190 ($11,220) | $52,278 ($66,782) | $25,691 ($10,150) | NA | $41,300 ($0) | NA | NA | $28,467 ($4,965) | NA | $61,171 ($84,633) | $9,220 ($575) | $6,681 ($2,008) | $811 ($0) | $25,852 ($20,125) |
| 53 | Other administration | /project | $470 ($285) | $1,678 ($2,786) | $10,852 ($13,991) | NA | $222 ($0) | $20,611 ($0) | NA | NA | NA | $2,710 ($0) | $817 ($1,342) | $5,252 ($1,008) | NA | $80 ($0) | $4,744 ($6,881) |
| **Category 5. Financial support for CDTi and surveillance in (post) conflict endemic areas** | | | | | | | | | | | | | | | | | |
| 54 | Support for CDTi and surveillance in (post) conflict endemic areas**p** | /endemic African regions | $1,052,363 (NA) | | | | | | | | | | | | | | |
| Note: if not specified below, data sources are project budgets.  **+** Equatorial Guinea  ***** Regional average across national averages  **^#^** The nationwide project had ten sub-project budgets available. To compare with projects in other countries, average unit costs per sub-project were shown.  **^%^** The nationwide project had seven sub-project budgets available. To compare with projects in other countries, average unit costs per sub-project were shown.  **^@^** There were two regional projects for which seven sub-project budgets were available. To compare with projects in other countries, average unit costs per sub-project were shown.  **a,b,c** Agriculture value added per person-day [9,10]  **d** 1) As a proxy for the costs of the microscopic diagnostic tools for *L.loa*, we used the costs of the epidemiological laboratory testing supplies (ID:41);  2) Source for delivery and administration of doxycycline: Wanji et al. 2009 [11]  **e** Budget for the training of community volunteers was used.  **f** Budget for the fuel support for supervisory visit to districts was used.  **g** Budget for the perdiem for health workers who train community drug distributors was used.  **h** Prices of 2 liters of distilled water, 200 glass slides, 3 instrument trays, 4 slide trays each holding 3 glass slides, liquid detergent, butane burning stove, dropper bottle, aluminium pressure sterilizer, cotton swabs soaked with alcohol, curved tweezer, holth punch, lancets, scissors, sterilizer forcepts. http://www.amazon.com Accessed on 17 August 2014.  **i** Budget for the training incentive for community drug distributors was used.  **j** Price of aspirator and plastic bottles. http://www.amazon.com Accessed on 17 August 2014.  **k** Budget for the transportation support for supervisory visit from health facility to villages was used.  **l** DHL service guide. http://www.dhlguide.co.uk Accessed on 17 August 2014.  **m** Budget for the perdiem for health workers who train community drug distributors was used.  **n** Prices of 1ml test tube, binocular microscope, blood slids, hypodermic syringe and needle, micro pipette with disposable tips, microtitration trays, saline solution, slide trays. http://www.amazon.com Accessed on 17 August 2014  **o** Daily wage of state-certified nurse in Burkina Faso[12] was used as a proxy for the daily wage of laboratory technicians in MSDC. For Ethiopia, Uganda, and Sudan that were identified to conduct the entomological laboratory testing in their own laboratories, we used the daily wage for technicians who conduct epidemiological laboratory testing, namely, $13, $8, and $15 (regional average), respectively (ID:40).  **p** Annual average funding for (post) conflict areas in Africa based on the APOC budget plan for 2008-2015 (annual average: $756,250) and Sightsavers’s strategic plan for 2011-2021 (annual average: $296,113) [13,14]. | | | | | | | | | | | | | | | | | |

**S4 Table. Agriculture value added per person-day for endemic African countries**

| **Country** | **Agriculture value added per person-day*** |
| --- | --- |
| Angola | $2.11 |
| Benin | $0.93 |
| Burkina Faso | $0.88 |
| Burundi | $0.39 |
| Cameroon | $0.92 |
| Central African Republic | $1.00 |
| Chad^ɸ^ | $0.57 |
| Congo, Dem. Rep. | $0.40 |
| Congo, Rep. | $0.41 |
| Côte d'Ivoire | $1.28 |
| Equatorial Guinea^†^ | $12.33 |
| Ethiopia | $0.87 |
| Gabon | $1.68 |
| Ghana | $1.45 |
| Guinea | $0.39 |
| Guinea-Bissau | $1.11 |
| Liberia | $0.62 |
| Malawi | $0.29 |
| Mali | $1.13 |
| Mozambique | $0.66 |
| Nigeria | $2.32 |
| Senegal | $0.66 |
| Sierra Leone | $1.38 |
| South Sudan^ǂ^ | $0.54 |
| Sudan | $1.80 |
| Tanzania | $0.64 |
| Togo | $0.69 |
| Uganda | $0.55 |
| Average (SD) | $1.36 ($2.22) |
| * To estimate daily agriculture value added per worker, agriculture value added per worker as percentage of GDP [9] was multiplied with 2012 GDP per capita [10], and was divided by 261 days.  ɸ,†,ǂ Agriculture value added per worker (% of GDP) for sub-Saharan Africa (developing only, 2012) was used, because national data were unavailable.  SD: standard deviation | |

**S5 Table. The ratio of community volunteers and of community health workers over population and population per district and per community for endemic African countries**

| **Country** | **Community volunteers over population** | **Community health workers over population** | **Population per district** | **Population per community** | **Source** |
| --- | --- | --- | --- | --- | --- |
| Angola | 1/275 | 1/2,458 | 70,460 | 910 | Project budgets |
| Benin | NA | NA | 51,106 | 564 | Sightsavers [14] |
| Burkina Faso | NA | NA | 171,150 | NA | Helen Keller International [15] |
| Burundi | 1/150 | 1/4,892 | 149,684 | 4,270 | Project budgets |
| Cameroon | 1/260 | 1/9,068 | 109,238 | 1,317 | Project budgets |
| CAR | 1/106 | 1/3,501 | 160,356 | 288 | Project budgets |
| Chad | 1/182 | 1/2,789 | 99,664 | 583 | Project budgets |
| Congo | 1/251 | 1/2,118 | 57,500 | 1,076 | Project budgets |
| Côte d`Ivoire | NA | NA | NA | NA | NA |
| DRC | NA | NA | NA | 646 | WHO/APOC [16] |
| Equatorial Guinea | 1/181 | 1/5,082 | 20,330 | 630 | Project budgets |
| Ethiopia | 1/188 | 1/14,930 | 40,156 | 396 | Project budgets |
| Gabon | NA | NA | 32,291 | NA | Direction Générale des Statistiques, Gabon [17,18] |
| Ghana | NA | NA | 36,060 | 654 | Ministry of health, Ghana [19] |
| Guinea | NA | NA | 312,617 | 304 | Institut National de la Statistique, the Republic of Guinea [20,21], Sightsavers [14] |
| Guinea-Bissau | NA | NA | 32,358 | 83 | Instituto Nacional de Estatística e Censos, Guinea-Bissau [22,23], Sightsavers [14] |
| Liberia | 1/196 | 1/2,920 | 165,043 | 696 | Project budgets |
| Malawi | 1/112 | 1/663 | 311,425 | 1,111 | Project budgets |
| Mali | NA | NA | 290,344 | 1,498 | Institut National de la Statistique, Mali [24,25], Sightsavers [14] |
| Mozambique | NA | NA | 173,837 | NA | Instituto Nacional de Estatistica, Mozambique [26,27] |
| Nigeria | 1/265 | 1/905 | 94,005 | 1,307 | Project budgets |
| Senegal | NA | NA | 300,182 | NA | Agence Nationale de la Statistique et de la Démographie (ANSD), Senegal [28,29] |
| Sierra Leone | NA | NA | 416,777 | 403 | Ministry of Health, Sierra Leone [30] |
| South Sudan | 1/379 | 1/6,180 | 114,929 | 958 | Project budgets |
| Sudan | NA | NA | NA | 676 | APOC [31] |
| Tanzania | 1/200 | 1/3,471 | 72,687 | 362 | Project budgets |
| Togo | NA | NA | 98,890 | 960 | Sightsavers [14] |
| Uganda | 1/47 | 1/1,266 | 134,919 | 638 | Project budgets |
| Average (SD) | 1/154 (0.0047) | 1/2,223 (0.0004) | 140,640 (107,370) | 884 (823) |  |

### Step 2. Costs at cost-item level


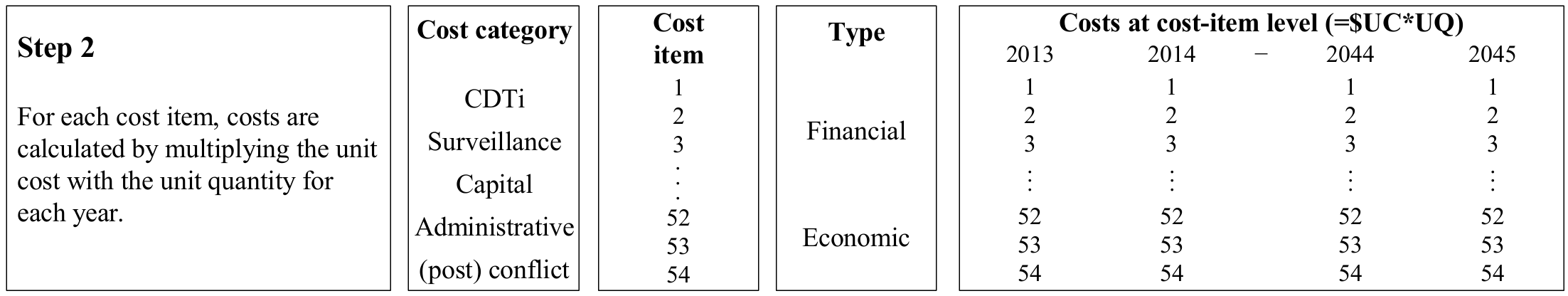


To estimate costs for each cost item from 2013 to 2045, we multiplied the unit cost with the unit quantity for each year. The unit quantity was adjusted for the relevant phases, that is, it was zero outside the relevant phases which are defined in S2 Table.

### Step 3. Classification into financial and economic costs


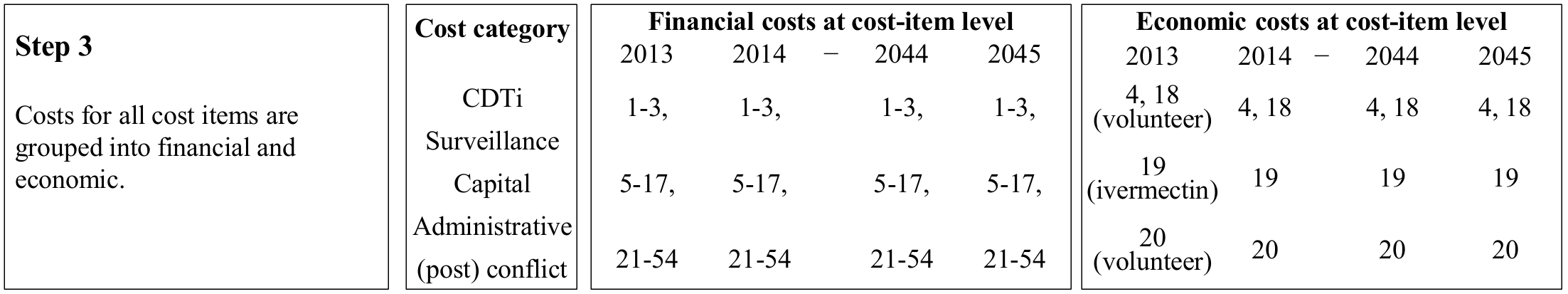


We grouped costs at cost-item level into financial and economic costs. Among the total 54 cost items, four cost items (ID: 4, 18, 19, 20) were for economic costs, and others were for financial costs.

### Step 4. Sub-classification of financial and economic costs


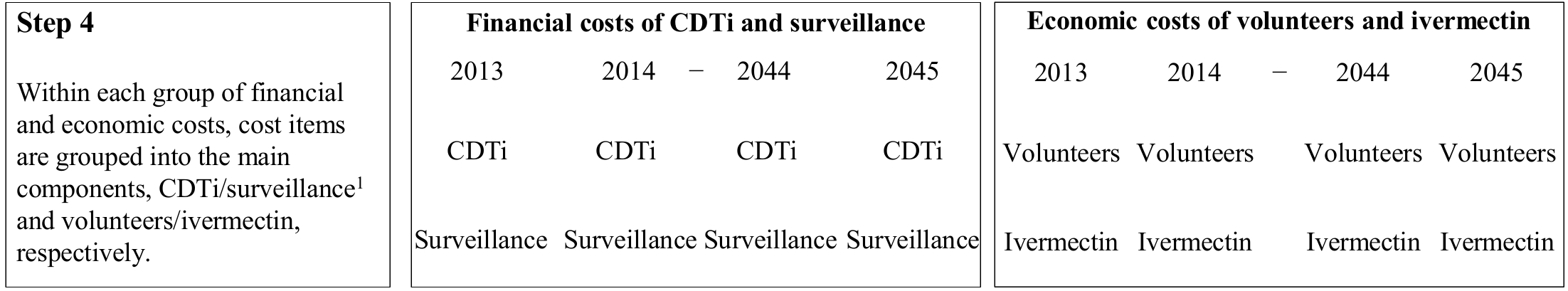

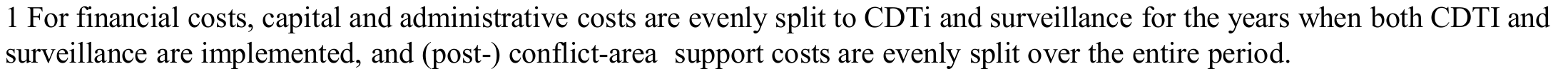


From an operational perspective, financial costs consist of those of CDTi and of surveillance. We grouped financial costs at cost-item level into those of CDTi and of surveillance based on the five categories. We evenly split the costs of capital goods and administration between CDTi and surveillance for years when both CDTi and surveillance were conducted; otherwise, allocated the costs to CDTi during the phase 1 and surveillance during the phases 2 and 3. We evenly split the support costs for (post) conflict areas between CDTi and surveillance for the entire time horizon. Economic costs consist of those of community volunteers and of donated ivermectin, and we grouped the economic costs at cost-item level into those two.

### Step 5. Total financial and economic costs


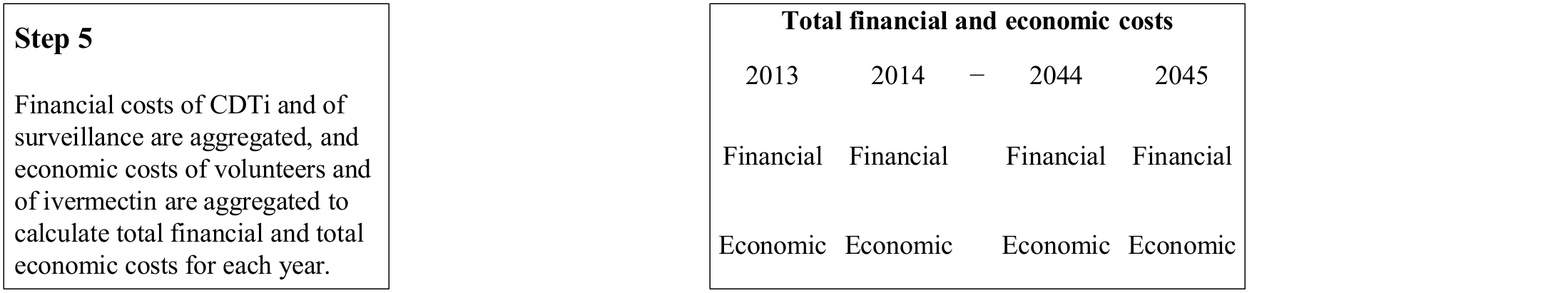


To estimate annual financial costs, we summed the annual financial costs of CDTi and surveillance (E1). To estimate annual economic costs, we summed the annual economic costs of community volunteers and donated ivermectin (E2).

$FC_{t}={FC}_{CDTi,t}+{FC}_{surveillance,t}$ (E1)

$EC_{t}=EC_{volunteer,t}+EC_{ivermectin,t}$ (E2)

FC_t_ : total financial costs for year *t*;

FC_CDTi_/_surveillance,t_ : financial costs of CDTi/surveillance for year *t*;

EC_t_ : total economic costs for year *t*;

EC_volunteer/ivermectin,t_ : economic costs of community volunteers/donated ivermectin for year *t*

### Step 6. Total costs for a project


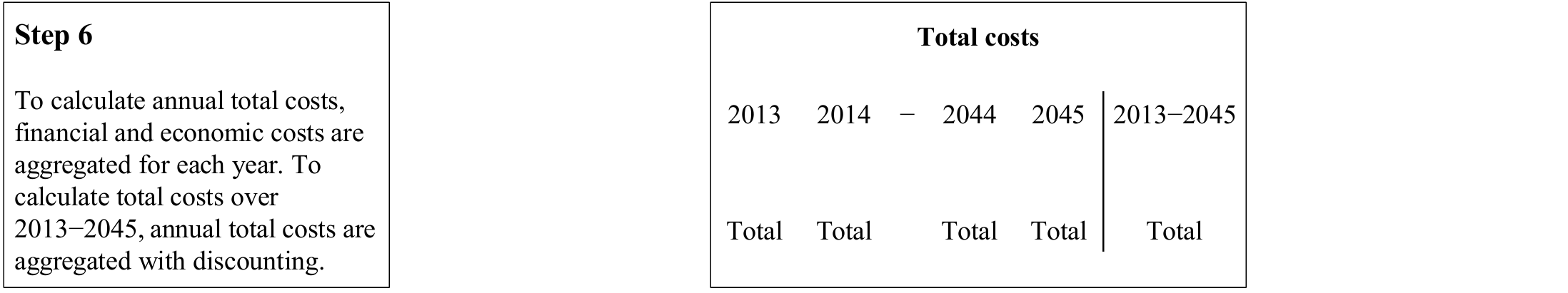


To calculate annual total costs for a project, we summed the annual financial and economic costs (E3).

$TC_{t}=FC_{t}+EC_{t}$ (E3)

TC_t_ : total costs for year *t*

To estimate cumulative costs over 2013 to 2045, we aggregated annual total costs with discounting (E4). The discount rate to account for time preference was 3%.

$${TC}_{2013-2045}=\sum_{t=2013}^{2045} \frac{TC_{t}}{\left( 1+r \right)^{\left( t-2012 \right)}}$$

(E4)

*r*: discount rate

## 3. Total costs for a scenario

To estimate total costs for a scenario, we aggregated costs at project level across the entire target projects.

# II. Uncertainty analysis

## 1. Selection of variables

We conducted sensitivity analysis to assess the robustness of results to parametric uncertainties. The parameters for the sensitivity analysis included cost items that had missing unit costs either for more than one third of total projects or total countries with budgets available, 22 of 67 projects or 5 of 14 countries (S6 Table). In addition to them, we also included the financial support costs for (post) conflict areas, as it was estimated based on only two sources, the strategic plans of APOC [13] and Sightsavers[14]. There were no missing data on the determinants of unit quantities for projects with budgets available. However, four determinants were time-variant; thus to assess the impact of time variation on total costs, we included them: population living in a project area, the number of required treatments (determined by population, treatment coverage linked to required treatment duration, and possible delay in starting and ending treatments), the number of required community volunteers (determined by population and the ratio of community volunteers over population), and the number of required community health workers (determined by population and the ratio of community health workers over population).

**S6 Table. Selected cost items for sensitivity analysis**

| **Cost items (ordered by no. of missing values)** | **Number of missing values** | |
| --- | --- | --- |
|  | **Country (/14)** | **Project (/67)** |
| Evaluation | 12 | 64 |
| Development of IEC material | 10 | 57 |
| Surveillance trip transportation | 6 | 38 |
| Monitoring | 6 | 33 |
| Delivery of fly samples from catching site to health facility | 5 | 35 |
| Data management | 5 | 26 |
| Management of severe adverse events | 4 | 30 |
| Salary top-ups (first 6 years) | 4 | 28 |
| Assistance for supervisory visits (7th year+) | 4 | 23 |

## 2. Statistical distributions

We applied statistical distributions to the selected variables considering the characteristics of variables with reference to standard practices [32].

We applied gamma distributions to the selected cost items and fitted them to available unit costs (S7 Table).

**S7 Table. Average and standard deviation of unit costs and distribution parameters**

| **Cost items** | **Average** | **Standard**  **deviation^†^** | **Gamma distribution^#^** | |
| --- | --- | --- | --- | --- |
|  |  |  | **Shape (k)** | **Scale (θ)** |
| **1 Evaluation** |  |  |  |  |
| Burundi | $3,585 | $358 | 100 | 36 |
| Malawi | $4,432 | $4,309 | 1 | 4,190 |
| Average | $4,008 | $599 | 45 | 90 |
| **2 Development of IEC material** |  |  |  |  |
| Ethiopia | $2,200 | $1,697 | 2 | 1,309 |
| Liberia | $4,532 | $99 | 2,107 | 2 |
| Nigeria | $1,889 | $1,566 | 1 | 1,299 |
| Uganda | $382 | $38 | 100 | 4 |
| Average | $2,250 | $1,716 | 2 | 1,308 |
| **3 Surveillance trip transportation** |  |  |  |  |
| Angola | $52 | $109 | >0 | 228 |
| Burundi | $14 | $18 | 1 | 24 |
| Cameroon | $32 | $56 | >0 | 100 |
| CAR | $9 | $2 | 13 | 1 |
| Chad | $6 | $1 | 100 | >0 |
| Ethiopia | $7 | $1 | 61 | >0 |
| Liberia | $21 | $3 | 49 | >0 |
| Nigeria | $30 | $44 | 1 | 63 |
| Average | $21 | $16 | 2 | 12 |
| **4 Monitoring** |  |  |  |  |
| Angola | $5,203 | $4,630 | 1 | 4120 |
| Burundi | $1,617 | $19 | 7593 | >0 |
| Cameroon | $10,482 | $7,449 | 2 | 5,293 |
| Chad | $1,468 | $676 | 5 | 312 |
| Ethiopia | $375 | $177 | 5 | 83 |
| Liberia | $1,735 | $17 | 10,034 | >0 |
| Malawi | $4,963 | $5,201 | 1 | 5,451 |
| Nigeria | $3,135 | $4,353 | 1 | 6,044 |
| Average | $3,622 | $3,259 | 1 | 2,933 |
| **5 Delivery of fly samples from catching site to health facility** |  |  |  |  |
| Angola | $5 | $3 | 2 | 3 |
| Burundi | $1 | >$0 | 100 | >0 |
| Cameroon | $6 | $8 | 1 | 10 |
| CAR | $12 | $9 | 2 | 7 |
| Chad | $6 | $1 | 100 | >0 |
| Ethiopia | $13 | $4 | 13 | 1 |
| Liberia | $5 | $5 | 1 | 5 |
| Malawi | $18 | $15 | 2 | 12 |
| Nigeria | $6 | $6 | 1 | 6 |
| Average | $8 | $5 | 2 | 3 |
| **6 Data management** |  |  |  |  |
| Angola | $1,400 | $140 | 100 | 14 |
| Burundi | $6,793 | $9,598 | 1 | 13,562 |
| Cameroon | $5,574 | $7,273 | 1 | 9,491 |
| Chad | $175 | $207 | 1 | 244 |
| Congo | $1,463 | $146 | 100 | 15 |
| Equatorial Guinea | $800 | $80 | 100 | 8 |
| Malawi | $1,344 | $540 | 6 | 217 |
| Nigeria | $975 | $2,220 | >0 | 5,053 |
| Uganda | $2,258 | $2,210 | 1 | 2,163 |
| Average | $2,309 | $2,287 | 1 | 2,265 |
| **7 Management of severe adverse events** |  |  |  |  |
| Angola | $1,360 | $381 | 13 | 107 |
| Burundi | $1,070 | $840 | 2 | 659 |
| Cameroon | $5,329 | $8,271 | >0 | 12,839 |
| Congo | $175 | $18 | 100 | 2 |
| Equatorial Guinea | $1,275 | $128 | 100 | 13 |
| Ethiopia | $2,465 | $247 | 100 | 25 |
| Liberia | $13,195 | $4,185 | 10 | 1,327 |
| Nigeria | $3,245 | $2,266 | 2 | 1,582 |
| South Sudan | $544 | $345 | 2 | 219 |
| Uganda | $1,277 | $128 | 100 | 13 |
| Average | $2,993 | $3,888 | 1 | 5,051 |
| **8 Salary top-ups (first 6 years)** |  |  |  |  |
| Angola | $14,708 | $8,284 | 3 | 4,666 |
| Burundi | $18,190 | $11,220 | 3 | 6,920 |
| Cameroon | $52,278 | $66,782 | 1 | 85,310 |
| CAR | $25,691 | $10,150 | 6 | 4,010 |
| Congo | $41,300 | $4,130 | 100 | 413 |
| Liberia | $28,467 | $4,965 | 33 | 866 |
| Nigeria | $61,171 | $84,633 | 1 | 117,094 |
| South Sudan | $9,220 | $575 | 257 | 36 |
| Tanzania | $6,681 | $2,008 | 11 | 604 |
| Uganda | $811 | $81 | 100 | 8 |
| Average | $25,852 | $20,125 | 2 | 15,668 |
| **9 Assistance for supervisory visits (7th year+)** |  |  |  |  |
| Angola | $4,000 | $1,414 | 8 | 500 |
| Burundi | $3,001 | $2,434 | 2 | 1,974 |
| Cameroon | $2,460 | $1,098 | 5 | 490 |
| Chad | $635 | $352 | 3 | 195 |
| Congo | $2,489 | $249 | 100 | 25 |
| Equatorial Guinea | $1,450 | $145 | 100 | 15 |
| Malawi | $1,253 | $655 | 4 | 342 |
| Nigeria | $1,702 | $1,480 | 1 | 1,287 |
| South Sudan | $1,500 | $150 | 100 | 15 |
| Uganda | $2,498 | $566 | 19 | 128 |
| Average | $2,099 | $981 | 5 | 459 |
| **10 Financial support for post-conflict areas (annual average)** |  |  |  |  |
| APOC | $756,250 | $7,563 | 100 | 7,563 |
| Sightsaver | $296,113 | $2,961 | 100 | 2,961 |
| † If there was only one data point, the standard deviation was assumed to be 10% of the data.  # Parameters of *Gamma(k, θ)* were estimated with a method of moments:  $shape \hat{k}=\frac{\bar{x^{2}}}{\bar{v}},scale \hat{\theta}=\frac{\bar{v}}{\bar{x}}$ $where \bar{x} is a sample mean, \bar{v} is a sample variance$ | | | | |

We used normal distributions to model the uncertainty about population growth rates from 2013 to 2045 for all endemic countries. We fitted the distribution to national low-high ranges[8], assuming the ranges to be the 95% confidence intervals.

To model the uncertainty about the number of treatments, we identified its determinants with reference to the study by Kim and colleagues [2]. The determinants were population living in a project area, treatment coverage, and the delay in starting and ending CDTi. We used normal distributions to model the uncertainty about population growth rates as described in the previous step. We used beta distributions to model the uncertainty about treatment coverage by fitting them to the treatment coverage data over 2010−2012 for APOC countries (source: APOC treatment database) (S8 Table). Projects in former OCP countries had no historical treatment coverage available, yet had the most recent year’s data. To estimate distribution parameters for the former OCP countries, we assumed that the standard deviation is 10% of treatment coverage. The change of treatment coverage was linked to the required duration of CDTi based on ONCHOSIM simulation results for the elimination and eradication scenarios. For the control scenario, the required duration of CDTi was extended for another 25 years if the treatment coverage decreased below the minimum required level, 65%. To model the uncertainty about the delay in starting and ending CDTi, we used a gamma distribution in which 90% of samples fall into the range of zero to five (shape=1, scale=2.25), assuming the delay can be as long as five years [2].

**S8 Table. Average and standard deviation of treatment coverage and distribution parameters**

| **Country** | **Average** | **Standard**  **deviation** | **Beta distribution^γ^** | |
| --- | --- | --- | --- | --- |
|  |  |  | **Alpha (α)** | **Beta (β)** |
| **APOC countries** |  |  |  |  |
| Angola | 67% | 0.11 | 12 | 6 |
| Burundi | 77% | 0.04 | 106 | 31 |
| Cameroon | 78% | 0.05 | 63 | 18 |
| CAR | 80% | 0.03 | 168 | 41 |
| Chad | 81% | >0.00 | 124,658 | 29,241 |
| Congo | 81% | 0.02 | 208 | 47 |
| DRC | 71% | 0.13 | 7 | 3 |
| Equatorial Guinea | 71% | >0.00 | 292,469 | 119,749 |
| Ethiopia | 79% | 0.04 | 78 | 21 |
| Liberia | 77% | 0.10 | 13 | 4 |
| Malawi | 83% | >0.00 | 5,113 | 1,066 |
| Nigeria | 80% | 0.04 | 83 | 21 |
| South Sudan | 60% | 0.12 | 9 | 6 |
| Sudan | 82% | 0.03 | 167 | 38 |
| Tanzania | 81% | 0.01 | 868 | 210 |
| Uganda | 75% | 0.11 | 10 | 3 |
| Average | 76% | 0.06 | 35 | 11 |
| **Former OCP countries** |  |  |  |  |
| Benin | 48% | 0.05 | 51 | 55 |
| Burkina Faso | 84% | 0.08 | 16 | 3 |
| Côte d’Ivoire | 84% | 0.08 | 16 | 3 |
| Ghana | 73% | 0.07 | 27 | 10 |
| Guinea | 73% | 0.07 | 27 | 10 |
| Guinea-Bissau | 73% | 0.07 | 27 | 10 |
| Mali | 73% | 0.07 | 27 | 10 |
| Senegal | 77% | 0.08 | 22 | 6 |
| Sierra Leone | 80% | 0.08 | 19 | 5 |
| Togo | 77% | 0.08 | 22 | 6 |
| Average | 74% | 0.10 | 13 | 5 |
| γ Parameters of *Beta(α, β)* were estimated using a method of moments:  $\hat{\alpha}=\bar{x}\left( \frac{\bar{x}\left( 1-\bar{x} \right)}{\bar{v}}-1 \right),\hat{\beta}=\left( 1-\bar{x} \right)\left( \frac{\bar{x}\left( 1-\bar{x} \right)}{\bar{v}}-1 \right), if \bar{v}<\bar{x}\left( 1-\bar{x} \right),$ $where \bar{x} is a sample mean, \bar{v} is a sample variance$ | | | | |

To model the uncertainty about the number of community volunteers and of community health workers, we identified their determinants, namely, population living in a project area and the ratio of community volunteers and of community health workers over population. We used normal distributions to model the uncertainty about population growth rates as described previously. We used beta distributions to model the uncertainty about the ratio of community volunteers over population and the ratio of community health workers over population by fitting them to relevant data (S9 Table).

For all selected variables, if there was no distribution at country level, we used distributions for the endemic regions which were estimated based on available national averages for endemic African countries.

**S9 Table. Average and standard deviation of the ratio of community volunteers over population and the ratio of community health workers over population and distribution parameters**

| **Country** | **Average** | **Standard deviation** | **Beta distribution^δ^** | |
| --- | --- | --- | --- | --- |
|  |  |  | **Alpha (α)** | **Beta (β)** |
| **1 Ratio of community volunteers over population** | | | | |
| Angola | 1/257 | 0.0029 | 2 | 429 |
| Burundi | 1/150 | 0.0005 | 164 | 24,462 |
| Cameroon | 1/260 | 0.0040 | 1 | 238 |
| CAR | 1/106 | 0.0019 | 25 | 2,600 |
| Chad | 1/182 | 0.0011 | 25 | 4,500 |
| Congo | 1/251 | 0.0008 | 25 | 6,225 |
| Equatorial Guinea | 1/181 | 0.0011 | 25 | 4,475 |
| Ethiopia | 1/188 | 0.0003 | 404 | 75,638 |
| Liberia | 1/196 | 0.0028 | 3 | 662 |
| Malawi | 1/112 | 0.0013 | 44 | 4,865 |
| Nigeria | 1/265 | 0.0028 | 2 | 472 |
| South Sudan | 1/379 | 0.0017 | 3 | 949 |
| Tanzania | 1/200 | 0.0010 | 25 | 4,950 |
| Uganda | 1/47 | 0.0155 | 2 | 85 |
| Average | 1/154 | 0.0047 | 2 | 294 |
| **2 Ratio of community health workers over population** | | | | |
| Angola | 1/2,458 | 0.0004 | 1 | 2,241 |
| Burundi | 1/4,892 | >0.0000 | 23 | 114,727 |
| Cameroon | 1/9,068 | 0.0003 | 0.2 | 1,602 |
| CAR | 1/3,501 | 0.0001 | 25 | 87,483 |
| Chad | 1/2,789 | 0.0001 | 25 | 69,676 |
| Congo | 1/2,118 | 0.0001 | 25 | 52,911 |
| Equatorial Guinea | 1/5,082 | >0.0000 | 25 | 127,009 |
| Ethiopia | 1/14,930 | 0.0001 | 1 | 21,589 |
| Liberia | 1/2,920 | 0.0001 | 6 | 18,393 |
| Malawi | 1/663 | 0.0006 | 7 | 4,688 |
| Nigeria | 1/905 | 0.0010 | 1 | 1,080 |
| South Sudan | 1/6,180 | 0.0001 | 4 | 24,603 |
| Tanzania | 1/3,471 | 0.0001 | 25 | 86,715 |
| Uganda | 1/1,266 | 0.0002 | 16 | 20,812 |
| Average | 1/2,223 | 0.0004 | 1 | 2614 |
| δ Parameters of *Beta(α, β)* were estimated using a method of moments:  $\hat{\alpha}=\bar{x}\left( \frac{\bar{x}\left( 1-\bar{x} \right)}{\bar{v}}-1 \right),\hat{\beta}=\left( 1-\bar{x} \right)\left( \frac{\bar{x}\left( 1-\bar{x} \right)}{\bar{v}}-1 \right), if \bar{v}<\bar{x}\left( 1-\bar{x} \right),$ $where \bar{x} is a sample mean, \bar{v} is a sample variance$ | | | | |

## 3. Simulation

We conducted one-way sensitivity analysis to examine the impact of parameters related to CDTi performance (treatment coverage, the delay in starting and ending CDTi), the selected cost items with high uncertainty, and discount rates (0%, 3%, 6%) on total costs. We also conducted multivariate PSA to examine the joint effects of uncertainties about all selected variables on total costs by running the micro-costing simulation 1,000 times using samples drawn from the distributions of the selected variables.

# III. Literature review

To find literature on regional elimination strategies for onchocerciasis in Africa, we used the PubMed (MEDLINE) database to search for documents in English and French, published between 2004 and 2014, with the following search terms: “onchocerciasis or river blindness” in title and “elimination or eradication” in abstract. We reviewed abstracts to determine relevance, and reviewed the full texts of selected documents. We also searched the bibliographies of identified references and the gray literature. We found two manuscripts on elimination strategies for endemic African regions: one a conceptual and operational framework of onchocerciasis elimination developed by APOC [33] and the other a manuscript on control, elimination, and eradication scenarios for onchocerciasis by Kim and colleagues [2] which was developed based on the former (S10 Table).

**S10 Table. Literature on regional elimination strategies in Africa**

|  | **Title** | **Authors** | **Published year** | **Reference** |
| --- | --- | --- | --- | --- |
| 1 | Conceptual and Operational Framework of Onchocerciasis Elimination with Ivermectin Treatment. | African Programme for Onchocerciasis Control | 2010 | [33] |
| 2 | Control, elimination, and eradication of river blindness: scenarios, timelines, and ivermectin treatment needs in Africa | Kim YE, Remme JHF, Steinmann P, Stolk WA, Roungou J, Tediosi F. | 2015 | [2] |

To estimate costs associated with potential elimination strategies proposed by Kim and colleagues, we searched literature on onchocerciasis intervention costs. Keating and colleagues searched literature on onchocerciasis intervention costs, published in English and French between 1990 and 2010, using PubMed (MEDLINE), EMBASE, and JSTOR databases with the following search terms: onchocerciasis, cost, cost–benefit, cost-effectiveness, economic, economics, internal rate of return, elimination, eradication, health systems, vertical, integration [34]. They selected publications after reviewing abstracts for relevance, and reviewed the full texts of the selected ones. They also searched the bibliographies of identified references and the gray literature. They found ten publications on costs for onchocerciasis treatment with ivermectin or doxycycline. We have extended the search period to include 2011−2015 using the same method, and found four more documents (S11 Table). Considering our objective was to estimate costs for potential elimination strategies in Africa using a micro-costing method, the identified publications were insufficient, because they focused on a limited number of countries, and many of them were outdated and lacked detailed data on resource utilization (e.g., unit costs and unit quantities). Also, all identified publications estimated costs for control strategies without regular surveillance.

**S11 Table. Literature on costs of onchocerciasis treatment with ivermectin or doxycycline in Africa**

|  | **Title** | **Authors** | **Published year** | **Country/region** | **Reference** |
| --- | --- | --- | --- | --- | --- |
| 1 | Ivermectin-based onchocerciasis control in Cameroon | Ngoumou P, Essomba RO, Godin C. | 1996 | Cameroon | [35] |
| 2 | Delivery systems and cost recovery in Mectizan treatment for onchocerciasis | Amazigo U, Noma M, Boatin BA, Etya'ale DE, Seketeli A, Dadzie KY. | 1998 | Endemic African regions | [36] |
| 3 | Ivermectin distribution using community volunteers in Kabarole district, Uganda | Kipp W, Burnham G, Bamuhiiga J, Weis P, Buttner DW. | 1998 | Uganda | [37] |
| 4 | The Mectizan (Ivermectin) Donation Program for Riverblindness as a Paradigm for Pharmaceutical Industry Donation Programs | Philip E.Coyne, David W.Berk. | 2001 | NA | [6] |
| 5 | Implementing community-directed treatment with ivermectin for the control of onchocerciasis in Uganda (1997–2000): an evaluation | Katabarwa MN, Habomugisha P, Richards FO, Jr. | 2002 | Uganda | [38] |
| 6 | Community-directed treatment with ivermectin in two Nigerian communities: an analysis of first year start-up processes, costs and consequences | Onwujekwe O, Chima R, Shu E, Okonkwo P. | 2002 | Nigeria | [39] |
| 7 | Economic evaluation of Mectizan distribution | Waters HR, Rehwinkel JA, Burnham G. | 2004 | Endemic African regions | [40] |
| 8 | Progress towards the elimination of onchocerciasis as a public-health problem in Uganda: opportunities, challenges and the way forward | Ndyomugyenyi R, Lakwo T, Habomugisha P, Male B. | 2007 | Uganda | [41] |
| 9 | Community-directed delivery of doxycycline for the treatment of onchocerciasis in areas of coendemicity with loiasis in Cameroon | Wanji S, Tendongfor N, Nji T, Esum M, Che JN, Nkwescheu A et al. | 2009 | Cameroon | [11] |
| 10 | Cost-effectiveness of triple drug administration (TDA) with praziquantel, ivermectin and albendazole for the prevention of neglected tropical diseases in Nigeria | Evans D, McFarland D, Adamani W, Eigege A, Miri E, Schulz J et al. | 2001 | Nigeria | [42] |
| 11 | African Programme For Onchocerciasis Control 1995-2015: model-estimated health impact and cost | Coffeng LE, Stolk WA, Zoure HG, Veerman JL, Agblewonu KB, Murdoch ME et al. | 2013 | Endemic African regions | [43] |
| 12 | The cost of annual versus biannual community-directed treatment of onchocerciasis with ivermectin: Ghana as a case study | Turner HC, Osei-Atweneboana MY, Walker M, Tettevi EJ, Churcher TS, Asiedu O et al. | 2013 | Ghana | [44] |
| 13 | Reaching the london declaration on neglected tropical diseases goals for onchocerciasis: an economic evaluation of increasing the frequency of ivermectin treatment in Africa | Turner HC, Walker M, Churcher TS, Osei-Atweneboana MY, Biritwum NK, Hopkins A et al. | 2014 | Endemic African regions | [45] |
| 14 | Onchocerciasis control in the Democratic Republic of Congo (DRC): challenges in a post-war environment | Makenga Bof JC, Maketa V, Bakajika DK, Ntumba F, Mpunga D, Murdoch ME et al. | 2015 | the Democratic Republic of Congo | [46] |

Reference List

1. WHO. APOC - community-directed treatment with ivermectin (CDTI) projects. WHO. 2008. <http://www.who.int/apoc/cdti/howitworks/en/> Accessed on 09 April 2015.

2. Kim YE, Remme JHF, Steinmann P, Stolk WA, Roungou JB, et al. Control, Elimination, and Eradication of River Blindness: Scenarios, Timelines, and Ivermectin Treatment Needs in Africa. PLoS Negl Trop Dis. 2015; 9: e0003664. doi:10.1371/journal.pntd.0003664.

3. WHO/APOC. Report of the twenty-ninth session of the technical consultative committee (TCC), Ouagadougou, 14-19 September 2009. DIR/COORD/APOC/REP/TCC29. WHO/APOC. 2010; 80-91.

4. OEPA. Guide for the detection of a potential recrudescence during the period of Post Treatment Surveillance (PTS). Onchocerciasis Elimination Program for the Americas. 2011. <http://www.oepa.net/Documentos/GuiaVEPT/Guide_Detection_Potential_Recrudescence_During_PTS_Englishversion.pdf> Accessed on 25 January 2015.

5. McFarland D, Menzies N, Njoumemi Z, and Onwujekwe O. Study of cost per treatment with ivermectin using the CDTI strategy. African Programme for Onchocerciasis Control (APOC). 2005.

6. Philip E.Coyne, David W.Berk. The Mectizan (ivermectin) donation program for river blindness as a paradigm for pharmaceutical industry donation programs. Washington: World Bank. World Bank. 2001. <http://apps.who.int/medicinedocs/documents/s17517en/s17517en.pdf> Accessed on 31 January 2015.

7. Goldman AS, Guisinger VH, Aikins M, Amarillo ML, Belizario VY, et al. National mass drug administration costs for lymphatic filariasis elimination. PLoS Negl Trop Dis. 2007; 1: e67. 10.1371/journal.pntd.0000067 [doi].

8. Population growth rates. World Population Prospects: the 2012 revision. UN. 2013. <http://esa.un.org/wpp/Excel-Data/population.htm> Accessed on 11 June 2015.

9. Agriculture value added (% of GDP), 2012. World Development Indicators. World Bank. 2014. <http://data.worldbank.org/indicator/NV.AGR.TOTL.ZS/countries> Accessed on 19 March 2015.

10. GDP per capita (current US$), 2012. World Bank national accounts data, and OECD National Accounts data files. World Bank. 2014. <http://data.worldbank.org/indicator/NY.GDP.PCAP.CD/countries> Accessed on 19 March 2015.

11. Wanji S, Tendongfor N, Nji T, Esum M, Che JN, et al. Community-directed delivery of doxycycline for the treatment of onchocerciasis in areas of co-endemicity with loiasis in Cameroon. Parasit Vectors. 2009; 2: 39. 1756-3305-2-39 [pii];10.1186/1756-3305-2-39 [doi].

12. McCoy D, Bennett S, Witter S, Pond B, Baker B, et al. Salaries and incomes of health workers in sub-Saharan Africa. Lancet. 2008; 371: 675-681. S0140-6736(08)60306-2 [pii];10.1016/S0140-6736(08)60306-2 [doi].

13. WHO/APOC. Addendum for the plan of action and budget 2008-2015. Ouagadougou: WHO/APOC. 2008.

14. Sightsavers. Elimination of onchocerciasis: ten-year strategic fast tracking plan in Sightsavers supported countries 2011-2021. Sightsavers. 2011. <http://www.sightsavers.net/about_us/publications/19377_0303_SS%20Oncho%20Report%20Lowres%20(4).pdf> Accessed on 31 March 2015.

15. Helen Keller International. Operational plan 2013 for neglected tropical diseases control in Burkina Faso: annual work plan (October 2012 - September 2013). Helen Keller International. 2012. <http://endinafrica.org/wp-content/uploads/2012/11/Burkina-Faso-Work-Plan-FY2013.pdf> Accessed on 06 February 2015.

16. APOC - Democratic Republic of the Congo. WHO/APOC. 2015. <http://www.who.int/apoc/countries/cod/en/> Accessed on 19 February 2015.

17. Total population, 2003 (last census). Direction Générale des Statistiques, the Gabonese Republic. 2015. <http://www.stat-gabon.com/> Accessed on 19 February 2015.

18. List of departments, 2003 (last census). Direction Générale des Statistiques, the Gabonese Republic. 2015. <http://www.stat-gabon.com/> Accessed on 19 February 2015.

19. Two-year strategic plan for integrated neglected tropical diseases control in Ghana 2007-2008. The Republic of Ghana, Ministry of Health, Ghana Health Service. 2006. <http://www.moh-ghana.org/UploadFiles/Publications/Plan%20for%20Pro-Poor%20Diseases120506091943.pdf> Accessed on 11 June 2015.

20. Total population, 2014. Institut National de la Statistique, the Republic of Guinea. 2015. <http://www.stat-guinee.org/> Accessed on 19 February 2015.

21. List of prefectures by region, 2014. Institut National de la Statistique, the Republic of Guinea. 2015. <http://www.stat-guinee.org/> Accessed on 19 February 2015.

22. Total population, 2009 (last census). Instituto Nacional de Estatística e Censos, Republic of Guinea-Bissau. 2015. <http://www.stat-guinebissau.com/> Accessed on 19 February 2015.

23. List of sectors by region, 2009 (last census). Instituto Nacional de Estatística e Censos, Republic of Guinea-Bissau. 2015. <http://www.stat-guinebissau.com/> Accessed on 19 February 2015.

24. Total population, 2009 (last census). Institut National de la Statistique, Mali. 2015. <http://www.instat-mali.org/> Accessed on 19 February 2015.

25. List of districts and circles by region, 2009 (last census). Institut National de la Statistique, Mali. 2015. <http://www.instat-mali.org/> Accessed on 19 February 2015.

26. Total population, 2007 (last census). Instituto Nacional de Estatistica, Mozambique. 2015. <http://www.ine.gov.mz/> Accessed on 19 February 2015.

27. List of districts, 2007 (last census). Instituto Nacional de Estatistica, Mozambique. 2015. <http://www.ine.gov.mz/> Accessed on 19 February 2015.

28. Total population, 2013. Agence Nationale de la Statistique et de la Démographie (ANSD), Republic of Senegal. 2015. <http://www.ansd.sn/> Accessed on 19 February 2015.

29. List of departments by region, 2013. Agence Nationale de la Statistique et de la Démographie (ANSD), Republic of Senegal. 2015. <http://www.ansd.sn/> Accessed on 19 February 2015.

30. National plan of action for intergrated control of onchocerciaisis, schistosomiais, soil transmitted helminthiasis and elimination of lymphatic filariasis in Sierra Leone 2006-2010. The Ministry Of Health and Sanitation of Sierra Leone. 2006. <http://files.givewell.org/files/Round2Apps/Cause1/GNNTDC/B/Sierra%20Leone%20-%20Plan%20of%20Action%20for%20integrated%20control-final.doc> Accessed on 06 February 2015.

31. APOC - country profiles: Sudan. WHO/APOC. 2015. <http://www.who.int/apoc/countries/sdn/en/> Accessed on 06 February 2015.

32. Andrew Briggs, Mark Sculpher, Karl Claxton. Making decision models probabilistic. In: Decision modelling for health economic evaluation. New York: Oxford University Press; 2006. pp. 77-120.

33. WHO/APOC. Conceptual and operational framework of onchocerciasis elimination with ivermectin treatment. WHO/APOC/MG/10.1. Ouagadougou: WHO/APOC. 2010. <http://www.who.int/apoc/oncho_elimination_report_english.pdf> Accessed on 25 January 2015.

34. Keating J, Yukich JO, Mollenkopf S, Tediosi F. Lymphatic filariasis and onchocerciasis prevention, treatment, and control costs across diverse settings: a systematic review. Acta Trop. 2014; 135: 86-95. S0001-706X(14)00100-4 [pii];10.1016/j.actatropica.2014.03.017 [doi].

35. Ngoumou P, Essomba RO, Godin C. Ivermectin-based onchocerciasis control in Cameroon. World Health Forum. 1996; 17: 25-28.

36. Amazigo U, Noma M, Boatin BA, Etya'ale DE, Seketeli A, et al. Delivery systems and cost recovery in Mectizan treatment for onchocerciasis. Ann Trop Med Parasitol. 1998; 92 Suppl 1: S23-S31.

37. Kipp W, Burnham G, Bamuhiiga J, Weis P, Buttner DW. Ivermectin distribution using community volunteers in Kabarole district, Uganda. Health Policy Plan. 1998; 13: 167-173.

38. Katabarwa MN, Habomugisha P, Richards FO, Jr. Implementing community-directed treatment with ivermectin for the control of onchocerciasis in Uganda (1997-2000): an evaluation. Ann Trop Med Parasitol. 2002; 96: 61-73. 10.1179/000349802125002419 [doi].

39. Onwujekwe O, Chima R, Shu E, Okonkwo P. Community-directed treatment with ivermectin in two Nigerian communities: an analysis of first year start-up processes, costs and consequences. Health Policy. 2002; 62: 31-51. S0168851001002263 [pii].

40. Waters HR, Rehwinkel JA, Burnham G. Economic evaluation of Mectizan distribution. Trop Med Int Health. 2004; 9: A16-A25. 10.1111/j.1365-3156.2004.01210.x [doi];TMI1210 [pii].

41. Ndyomugyenyi R, Lakwo T, Habomugisha P, Male B. Progress towards the elimination of onchocerciasis as a public-health problem in Uganda: opportunities, challenges and the way forward. Ann Trop Med Parasitol. 2007; 101: 323-333. 10.1179/136485907X176355 [doi].

42. Evans D, McFarland D, Adamani W, Eigege A, Miri E, et al. Cost-effectiveness of triple drug administration (TDA) with praziquantel, ivermectin and albendazole for the prevention of neglected tropical diseases in Nigeria. Ann Trop Med Parasitol. 2011; 105: 537-547. 10.1179/2047773211Y.0000000010 [doi].

43. Coffeng LE, Stolk WA, Zoure HG, Veerman JL, Agblewonu KB, et al. African Programme For Onchocerciasis Control 1995-2015: model-estimated health impact and cost. PLoS Negl Trop Dis. 2013; 7: e2032. 10.1371/journal.pntd.0002032 [doi];PNTD-D-12-00318 [pii].

44. Turner HC, Osei-Atweneboana MY, Walker M, Tettevi EJ, Churcher TS, et al. The cost of annual versus biannual community-directed treatment of onchocerciasis with ivermectin: Ghana as a case study. PLoS Negl Trop Dis. 2013; 7: e2452. 10.1371/journal.pntd.0002452 [doi];PNTD-D-13-00304 [pii].

45. Turner HC, Walker M, Churcher TS, Osei-Atweneboana MY, Biritwum NK, et al. Reaching the london declaration on neglected tropical diseases goals for onchocerciasis: an economic evaluation of increasing the frequency of ivermectin treatment in Africa. Clin Infect Dis. 2014; 59: 923-932. ciu467 [pii];10.1093/cid/ciu467 [doi].

46. Makenga Bof JC, Maketa V, Bakajika DK, Ntumba F, Mpunga D, et al. Onchocerciasis control in the Democratic Republic of Congo (DRC): challenges in a post-war environment. Trop Med Int Health. 2015; 20: 48-62. 10.1111/tmi.12397 [doi].
