# Supplementary figures and images for: Financial and Economic Costs of the Elimination and Eradication of Onchocerciasis (River Blindness) in Africa
Source: PLoS Negl Trop Dis. 2015 Sep 11;9(9):e0004056. doi: 10.1371/journal.pntd.0004056 (PMC4567329; doi:10.1371/journal.pntd.0004056)

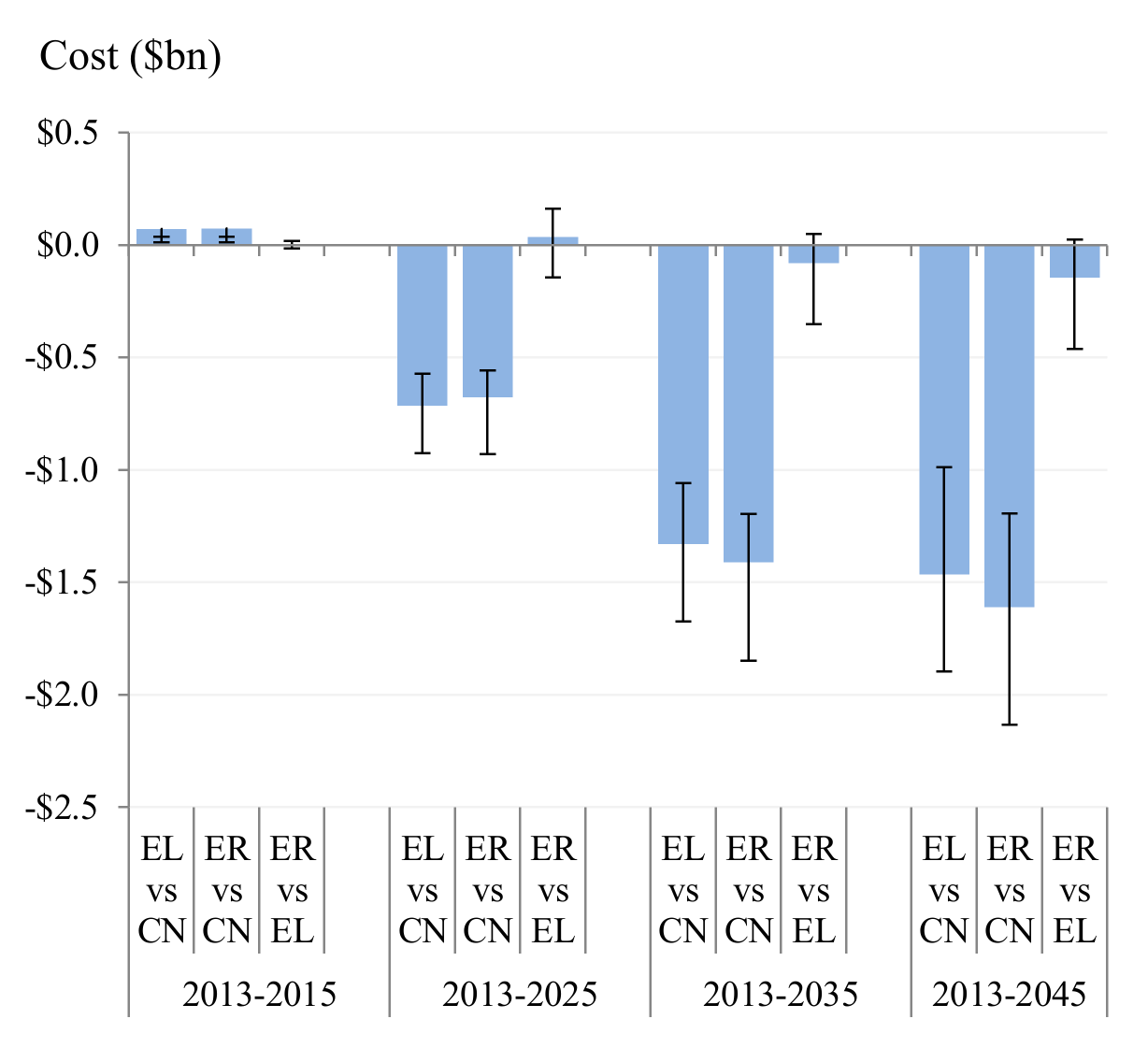

Supplement: S1 Fig — CN: control scenario, EL: elimination scenario, ER: eradication scenario (TIF) [file pntd.0004056.s002.tif]

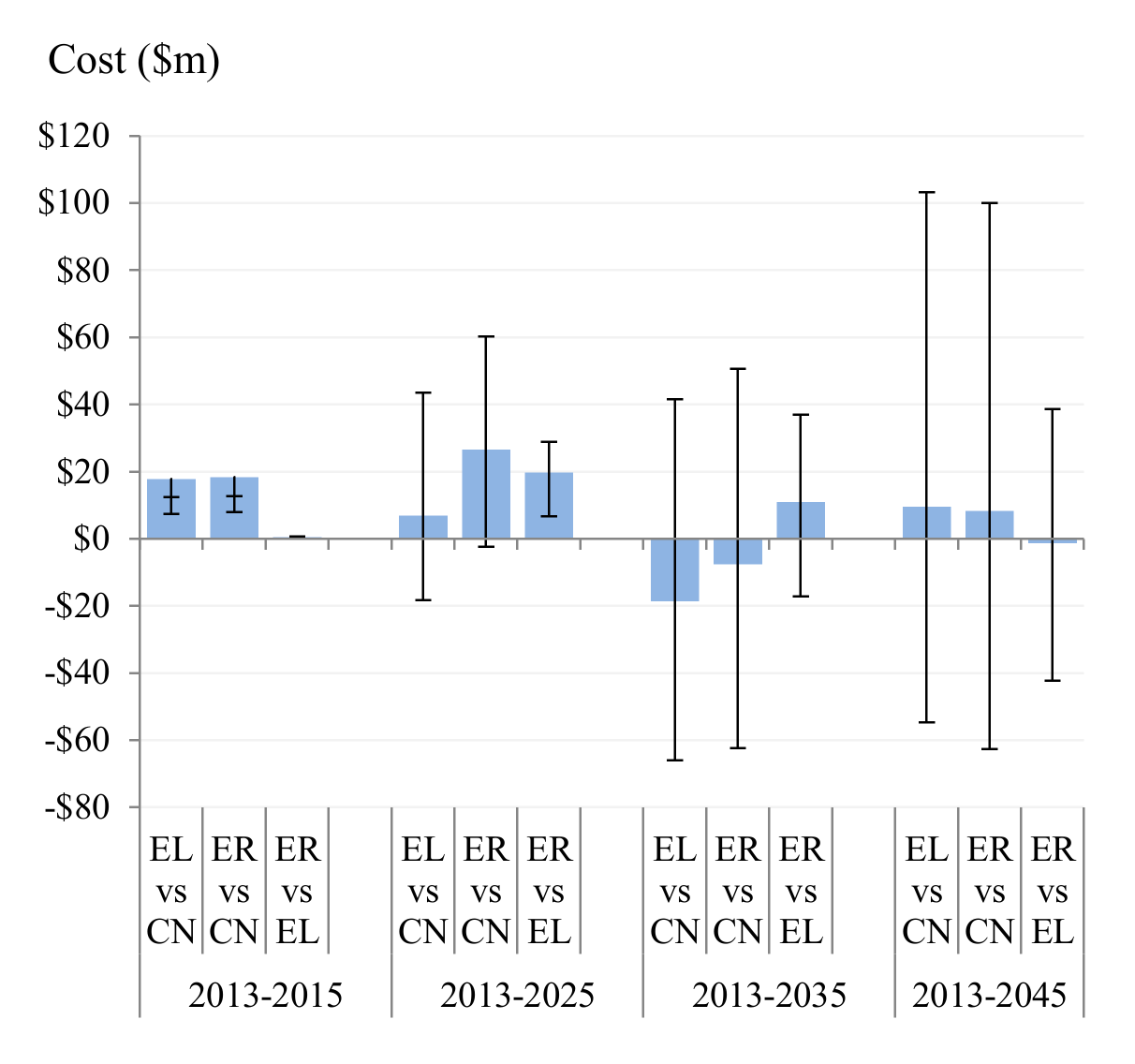

Supplement: S2 Fig — CN: control scenario, EL: elimination scenario, ER: eradication scenario (TIF) [file pntd.0004056.s003.tif]

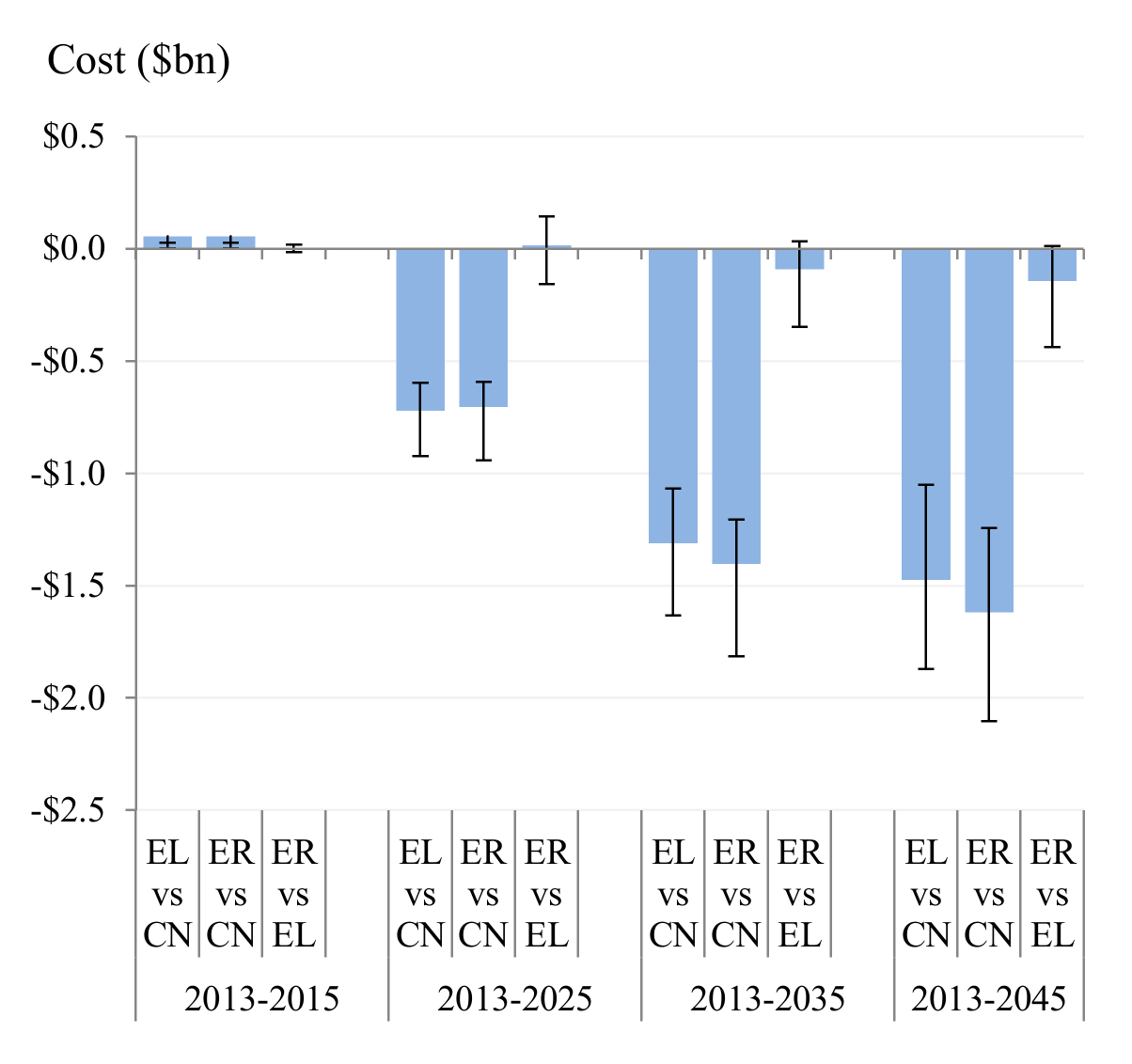

Supplement: S3 Fig — CN: control scenario, EL: elimination scenario, ER: eradication scenario (TIF) [file pntd.0004056.s004.tif]
